# A Comparison of Two Types of Rabbit Antithymocyte Globulin Induction Therapy in Immunological High-Risk Kidney Recipients: A Prospective Randomized Control Study

**DOI:** 10.1371/journal.pone.0165233

**Published:** 2016-11-17

**Authors:** F. Burkhalter, S. Schaub, Ch. Bucher, L. Gürke, A. Bachmann, H. Hopfer, M. Dickenmann, J. Steiger, I. Binet

**Affiliations:** 1 Clinic for Transplant Immunology and Nephrology, University Hospital Basel, Basel, Switzerland; 2 Department of Vascular and Transplant Surgery, University Hospital Basel, Basel, Switzerland; 3 Department of Urology, Basel University Hospital, Basel, Switzerland; 4 Institute for Pathology, University Hospital Basel, Basel, Switzerland; 5 Nephrology and Transplantation Medicine, Kantonsspital St Gallen, St Gallen, Switzerland; Ospedale San Raffaele, ITALY

## Abstract

**Background:**

Induction treatment with rabbit polyclonal antithymocyte globulins (ATGs) is frequent used in kidney transplant recipients with donorspecific HLA antibodies and shows acceptable outcomes. The two commonly used ATGs, Thymoglobulin and ATG-F have slightly different antigen profile and antibody concentrations. The two compounds have never been directly compared in a prospective trial in immunological high-risk recipients. Therefore we performed a prospective randomized controlled study comparing the two compounds in immunological high-risk kidney recipients in terms of safety and efficacy.

**Methods:**

Immunological high-risk kidney recipients, defined as the presence of HLA DSA but negative CDC-B and T-cell crossmatches were randomized 1:1 to receive ATG-F or Thymoglobulin. Maintenance immunosuppressive therapy consisted of tacrolimus, mycophenolate mofetil and steroids.

**Results:**

The per-protocol analysis included 35 patients. There was no immediate infusion reaction observed with both compounds. No PTLD or malignancy occurred during the follow-up in both groups. The incidence of viral and bacterial infections was similar in both groups (p = 0.62). The cumulative incidence of clinical and subclinical antibody mediated allograft rejection as well as T-cell mediated allograft rejection during the first year between ATG-F and Thymoglobulin was similar (35% versus 19%; p = 0.30 and 11% versus 18%; 0.54 respectively). The two-year graft function was similar with a median eGFR of 56 ml/min/1.73m^2^ (range 21–128) (ATG-F-group) and 51 ml/min/1.73m^2^ (range 22–132) (Thymo-group) (p = 0.69).

**Conclusion:**

We found no significant differences between the compared study drugs for induction treatment in immunological high-risk patients regarding safety and efficacy during follow-up with good allograft function at 2 years after transplantation.

## Introduction

During the last decade immunological risk stratification in kidney transplant patients had made eminent progress due to new methods used to identify HLA alloantibodies. It is known that such donor-specific HLA antibodies are associated with early antibody mediated allograft rejection and are the most important predictor of risk for rejection, in contrast to the traditional risk factors (high panel reactive antibodies, re-transplantation and deceased donor grafts)[1;2]. High level of HLA-DSA associated with a positive complement-dependent cytotoxicity crossmatch (CDC-XM) is considered as a contraindication for renal transplantation in most transplant centres. Patients with HLA-DSA detectable only by single HLA-antigen flow beads (SAFB) but with a negative CDC-XM are regarded as immunological risk patients for transplantation, but not considered as a contraindication. Several studies have reported reasonable outcomes in such patients with an induction treatment with polyclonal antithymocyte globulins (ATGs) and intravenous immunoglobulins (IVIG) [3–6]. Two of the compounds commonly used as antithymocyte induction treatment in these studies were ATG-Fresenius (ATG-F) and Thymoglobulin (Thymo). Both compounds are polyclonal antithymocyte IgG antibodies derived from rabbits after immunisation with a T-lymphoblast line (Jurkat cell line) in case of ATG-F and with human thymocytes in case of Thymo. ATGs are efficiently depleting T-cells and other leucocytes through various mechanisms (complement-dependent and cell-mediated cytotoxicity or via apoptosis induction). In addition other immunomodulatory effects contribute as well to the benefit of ATGs treatment. More than 40 leukocyte surface molecules are known to serve as antithymocyte antigens. There are quantitative differences of antibody concentrations against these different antigens between ATG-F and Thymo as well as small differences in their antigen profile [7].

When using ATGs as an induction treatment, there are concerns of short and long-term side effects in terms of malignancies, especially post-transplant lymphoproliferative diseases (PTLD) as well as for infectious complications. Further concerns are drug related early side effects. Both compounds are known to cause anaphylactic reactions and serum sickness as well as hematological side effects (thrombocytopenia, agranulocytosis and anemia). As both compounds are not similar in terms of antibody concentrations and their antibody profile, there might be as well a difference in their side effect profile. So far there are only retrospective studies comparing ATG-F and Thymo in terms of safety and efficacy in kidney transplantation. The aim of our prospective randomized controlled study in immunological high-risk kidney recipients was to assess whether safety and efficacy of the two compounds are comparable.

## Materials and Methods

This is a multicenter (University Hospital Basel and Kantonsspital St. Gallen) 1:1 randomized comparative open labelled study comparing safety and efficacy of two rabbit antithymocyte globulins compounds (ATG-Fresenius and Thymoglobulin) in immunological high-risk patients. Patients were included between November 2008 and February 2013. Inclusion criteria were adult recipients with a high immunological risk defined by the presence of at least one HLA donor-specific antibody (class I and/or II) detected by SAFB and with a negative T-cell and B-cell CDC-XM. Exclusion criteria were recipient age < 18 years, ABO-incompatible, white blood cells <3000/μl, thrombocytopenia <75000/μl, EBV risk constellation (recipient EBV negative and donor positive) and significant liver disease (defined as having ASAT(SGOT) and/or ALAT(SGPT) levels greater than 3 fold the upper value of the normal range). All patients gave written informed consent prior to randomisation. Patients were randomly assigned prior to transplantation by computer-generated selection. The computer-generated selection was provided by an independent company (Psy consult scientific services, Frankfurt, Germany). Numbered randomisation envelopes were provided for each center. After randomisation the investigators were not blinded to treatment group. The protocol was approved by the Institutional review boards of the University of Basel Switzerland and of the Kantonsspital St. Gallen Switzerland. The trial was registered in the ClinicalTrials.gov database (NCT00861536).

### Immunosuppression protocols

Patients who were randomized in the ATG-F-group received an induction therapy consisting of ATG-F (ATG-Fresenius, Fresenius Medical Care, Switzerland) 9 mg/kg body weight prior to reperfusion of the allograft, followed by 3 mg/kg body weight/d on day 1–4. Patients in the Thymo-group received an induction therapy consisting of Thymoglobulin (Thymoglobuline, Genzyme Polyclonals S.A.S., 69280 Marcy L’Etoile, Frankreich) 1.5mg/kg body weight prior to reperfusion of the allograft followed by 1.5mg/kg body weight on day 1–3. Doses of ATG-F and Thymoglobulin had to be reduced to half the dose when WBC count was 2000–3000 x 10e9/l and /or thrombocyte count was 50`000–75`000 x 10e9/l. In case of WBC count < 2000 x 10e9/l and/or thrombocyte count < 50`000 x 10e9/l ATG-F or Thymoglobulin had to be withheld.

Patient in both groups received intravenous immunglobuline (IVIG) 0.4g/kg body weight prior to reperfusion of the allograft and on day 1–4 (total dose 2g/kg body weight). Maintenance immunosuppression in both groups consisted of tacrolimus (Prograf, Astellas, Wallisellen, Switzerland), mycophenolate-mofetil (Cellcept, Roche, Basel, Switzerland) and prednisone. Target tacrolimus trough levels were 10–12 ng/ml for the first month, 8–10 ng/ml for months 2–3, 6–8 ng/ml for months 4–6 and 4–6 ng/ml thereafter. Initial mycophenolate-mofetil was 1000mg twice daily with the target trough level above 2 ug/ml. Pulse steroids were given from day 0–2 (500 mg methylprednisolon before the first dose of IVIG and an additional pulse of 500mg intraoperative, 500mg on day 1, 250mg day 2) and there after prednisone orally was started with a dose of 0.5mg/kg body weight/d and tapered to 0.1 mg/kg body weight by month 3 posttransplant. All patients received Pneumocystis jiroveci prophylaxis with Co-trimoxazole and in case of CMV-Risk (D+/R-, D+/R+, D-/R+) a prophylaxis with Valgancyclovir (VGC) for at least 3 months.

### Primary and secondary endpoints

The primary endpoints of the trial were safety parameters. Incidence of early study drug related side effects (anaphylaxis, serum sickness, fever >38°, chills, nausea/vomiting or erythema), early severe hematological side effects (leucocytopenia with white blood cells <3000/ul, severe thrombocytopenia <50’000/ul) during the first 4 weeks after transplantation. All adverse event/toxicity were assessed daily immediately posttransplant while patients were hospitalized, then postoperative day 14, 21, 28, 61, 90,180 and 365. Incidence of PTLD and malignancy were assessed at the final visit after 24 months of follow-up.

Secondary endpoints were incidence of acute rejection episodes and type (cellular or humoral) of rejection in protocol and clinically indicated biopsies, delayed graft function defined as requiring hemodialysis during the first week post-transplant due to inadequate allograft function, graft function (GFR and proteinuria), graft and patient survival, infectious complication during the 24 months follow-up. Effect of antithymocyte induction on peripheral T- (CD3+) and B- (CD19+) cell count were evaluated after 6 and 12 months.

### Typing of HLA-antigens

All donors and recipients were typed for HLA-A/B/DRB1/DQB1 antigens by serology and DNA-based methods. If a potential donor-specific HLA-Cw or HLA-DPB1 antibody was present, HLA-Cw and/or HLA-DPB1 antigens of the donor were determined by DNA-based typing methods.

### Detection of HLA-antibodies and assignment as HLA-DSA

All sera were tested for class I (i.e. HLA-A/B/Cw) and class II (i.e. HLA-DR/DQ/DP) HLA-antibodies using SAFB on a Luminex platform (LabScreen, One Lambda, Canoga Park, CA). A positive result was defined as a baseline normalized mean fluorescence intensity (MFI) > 500. HLA-DSA`s were determined by comparison of the HLA-antibody specificities of the recipient with the HLA-typing of the donor and included HLA-A/B/Cw/DRB1/DQB1/DPB1 loci. For every individual HLA DSA, the reported strength is based on the MFI of one SAFB. In case of more than one HLA-DSA against different HLA-antigens, the cumulative strength was calculated by adding the individual MFI values.

### CDC-XM assay

T- and B cells were isolated using immunomagnetic beads (Dynabeads, Dynal Biotech, Oslo, Norway). One uL of donor T and B cells was incubated with 1 uL of recipient sera for 30 and 40 min, respectively. Five uL rabbit complement and staining solution were added and incubated for 45 min. T- and B-cell CDC-XM were considered positive when the observer cell death exceeded 10% above background.

### Diagnosis of rejection

Clinically indicated allograft biopsies were performed when serum creatinine deteriorated by more than 20% or allograft function was not reaching the expected range during the first 4 weeks after transplantation. Within the current practice of our centers in patients with immunological risk, protocol biopsies were performed at day 7, month 3 and 6 post-transplant. Biopsy specimens (two cores obtained with a 16 gauge needle) were evaluated by light microscopy and immunofluorescence for C4d staining. Findings were graded according to the Banff 2007 classification.

### Sample Size

There are no prospective collected data available in terms of safety and efficacy comparing both compounds. So any sample size calculation was not possible due to lack of any data at the time of initiation of the study.

### Statistics analysis

We used JMP software version 12 (SAS Institute Inc.,Cary,NC) for statistical analysis. For categorical data, Fisher`s exact test or Pearson`s chi-square test were used. Normally distributed continuous data were analysed by Student`s *t*-test. For nonparametric continuous data, the Wilcoxon rank-sum test was used. Survival analysis was performed by the Kaplan-Meier method and groups compared using the log rank-test. A p-value < 0.05 was considered to indicate statistical significance.

## Results

### Patient characteristics

**Fig 1:**
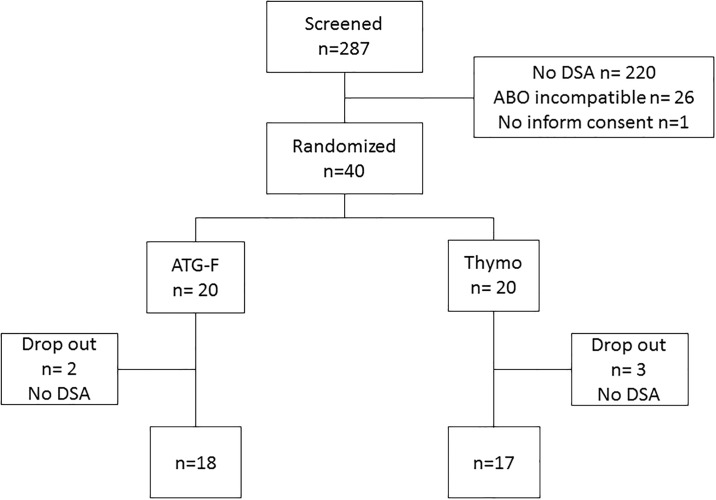
Patient Flow Chart.

Between November 2008 and February 2013 all patients who underwent kidney transplantation were prospectively screened for eligibility to participate in the study. A total of 40 adult patients were finally randomised in the study (Fig 1). Baseline characteristics of the two groups are detailed in Table 1. Five Patients (two patients in the ATG-F-group and three patients in the Thymo-group) were excluded after initial randomisation as further donor HLA typing of HLA-DQ, HLA-Cw, HLA-DPβ locus by SSP-DNA typing during the first 24h post-transplant revealed no evidence of DSA. There was no further exclusion in the follow-up, thus 35 patients remained for study analysis. The two groups were not different regarding donor parameters, HLA-matching, prior renal transplants, number and class of HLA-DSA as well as total mean fluorescence intensity (MFI) of HLA-DSA before transplantation.

**Table 1:** Baseline characteristics.

	ATG-F (n = 18)	Thymo (n = 17)	p-level
**Recipient**			
Gender, females (%)	7 (39)	7 (41)	1.00
Age, median (range)	55 (36–69)	52 (28–70)	0.31
Reason for ESRD			
autoimmune disease, n	3	3	
vascular/diabetic, n	3	3	
IgA nephritis, n	3	3	
ADPKD, n	7	3	
other reason, n	2	5	
**Donor**			
Deceased donor, n (%)	14 (78)	12 (71)	0.71
Extended criteria donor, n	6	5	
Non heart beating donor, n	0	0	
Gender, female (%)	9 (50)	11 (65)	0.50
Age, median (range)	55 (8–70)	57 (14–69)	0.91
Cold ischemia time(min), median (range)	436 (76–1200)	450 (49–1220)	0.82
**HLA-mismatches**			
1/2/3/4/5/6	1/2/3/7/4/1	2/4/6/3/2/0	0.39
**Number of Tx,**			
1/2/3/4	11/7/0/0	5/7/4/1	0.07
1 vs ≥ 2	11/7	5/12	0.06
**Number of DSA, n with 1/2/3/4 DSA**	10/5/2/1	13/3/1/0	0.53
**Class of DSA**			
Class I, n (%)	9 (50)	8 (47)	1.00
Class II, n (%)	5 (28)	7 (41)	0.49
Class I+II, n (%)	4 (22)	2 (12)	0.66
**Cumulative strength of DSA (MFI)**			
median (range)	3331 (774–28549)	4074 (880–33112)	0.90
**CMV**			
CMV D+/R-, n (%)	4 (22)	4 (23)	1.00
CMV D+/R+, D-/R+, n (%)	10 (56)	10 (59)	1.00
CMV D-/R-, n (%)	4 (22)	3 (18)	1.00
**EBV R+, n (%)**	18 (100)	17 (100)	1.00

### Side effects

We observed no immediate infusion reaction (anaphylaxis, fever, chill, nausea/vomiting, blood pressure drop or erythema) in both groups. In addition not a single serum sickness appeared during the treatment in the whole study population. Overall there were no dose reduction or withdrawal of ATGs needed due to an adverse event or hematological changes and so all the patients received the full dose of ATGs. Other safety issue (PTLD, malignancy) were not observed during the 24 months follow-up.

One serious adverse event occurred in the ATG-F-group. The patient developed disseminated intravascular coagulation (DIC) with severe bleeding 2 hours after the end of transplant surgery and 3 hours after the application of first dose of ATG-F. The complication led to primary allograft failure and the allograft had to be removed due to ongoing bleeding.

### Graft and patient survival

**Fig 2:**
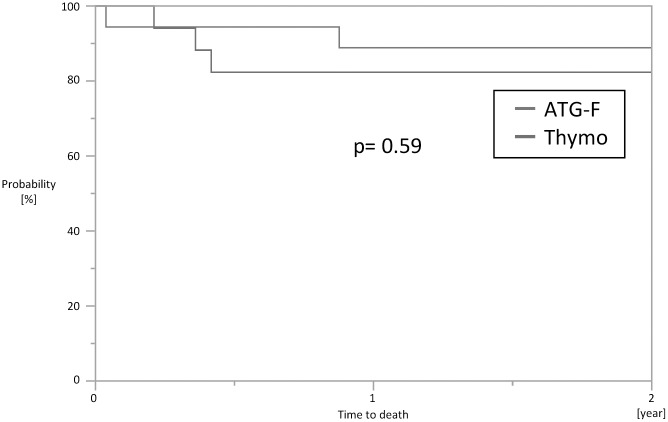
Patients survival.

The one-year and two-year allograft survival was equal between the groups (p = 0.33) with total only one allograft failure due to primary non-function in the ATG-F-group. After nephrectomy histological examination revealed no signs of acute AMR, but very severe tubular necrosis. Two patient in the ATG-F-group and three patients in the Thymo-group died during the first year after transplantation. Three patients died due to a sudden cardiac death. All three patients had known coronary heart disease and longstanding diabetes and received their second transplant. Another patient died after reanimation with cerebral hypoxia and one patient committed suicide (p = 0.59) (Fig 2).

### Hematological side effects

**Fig 3:**
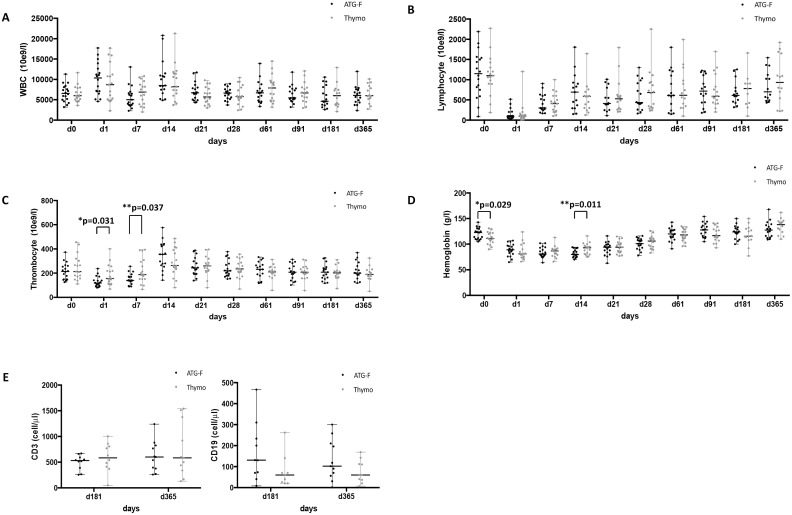
Hematological values during follow-up (A) Median white blood cells (WBC) count, (B) Median lymphocyte count, (C) Median thrombocyte count, (D) Median haemoglobin, (E) Mediant T- (CD3+) and B- (CD19+) cell count.

Hematological changes in both groups were as follows: White blood cells (WBC) counts were similar in both groups at any time of the whole study period (Fig 3A). Two patients, one in each group had WBC counts below 3000/ul at day 7. There was no difference in median lymphocyte count after the first dose of the ATGs (ATG-F or Thymo) (p = 0.75) and during follow-up (Fig 3B). The median T cell count was not different between the groups after 6 and 12 months (p = 0.36 and p = 0.84 respectively) as well as the median B cell count after 6 and 12 months (p = 0.34 and p = 0.52 respectively) (Fig 3E). But we observed a lower median thrombocyte count at day 1 and day 7 in the ATG-F-group compared in the Thymo-group (p = 0.0306 and 0.037) (Fig 3C). Median hemoglobin at baseline was significant different between the groups (p = 0.029). But after surgery at day 1 and day 7 there was no difference in median hemoglobin (p = 0.28 and 0.70 respectively) (Fig 3D). Also median hemoglobin drop between day 0 and day 1 was not different between the groups (p = 0.388). In both groups nearly half of the patients (44% and 41% of patients respectively) required blood transfusion during the first two weeks (p = 1.00). At day 14 we observed a significant lower median hemoglobin level in the ATG-F-group (p = 0.011) (Fig 3D). Thereafter median hemoglobin was similar in both groups.

### Infectious side effects

There was no difference in the incidence of bacterial infections after transplantation in both groups (p = 0.62) (Table 2). CMV Replication was seen in total in 6/35 patients at risk. There was no difference for CMV-Replication between the two groups (p = 0.4) (Table 2). CMV Replication did not lead to any CMV disease, as all the patients were treated with VGC as soon as replication was observed. EBV Replication was significantly more frequent in the ATG-F-group compared to the Thymo-group (p = 0.0455). But during long term follow-up and standard reduction of immunosuppressive therapy EBV replication disappeared in all patients.

**Table 2:** Infection diseases.

	ATG-F (n = 18)	Thymo (n = 17)	p level
**Bacterial infection overall**			0.62
0 episode	8	10	
1 episode	6	5	
>1 episode	4	2	
**Type of bacterial infection**			
UTI			
0 episode	12	13	0.81
≥1 episode	6	4	
Pyelonephritis	2	1	0.49
Wound infection	1	2	0.60
Other bact. Infections	5	2	0.55
**Fungal infection**	3	1	0.60
**CMV Replication (pos. PCR), n (at risk)**	2 (14)	4 (14)	0.40
**EBV Replication (pos. PCR), n (at risk)**	5 (18)	0 (17)	**0.0455**
**Herpesvirus (HSV and VZV)**	2	1	1.00

### Rejection episodes and allograft function

**Fig 4:**
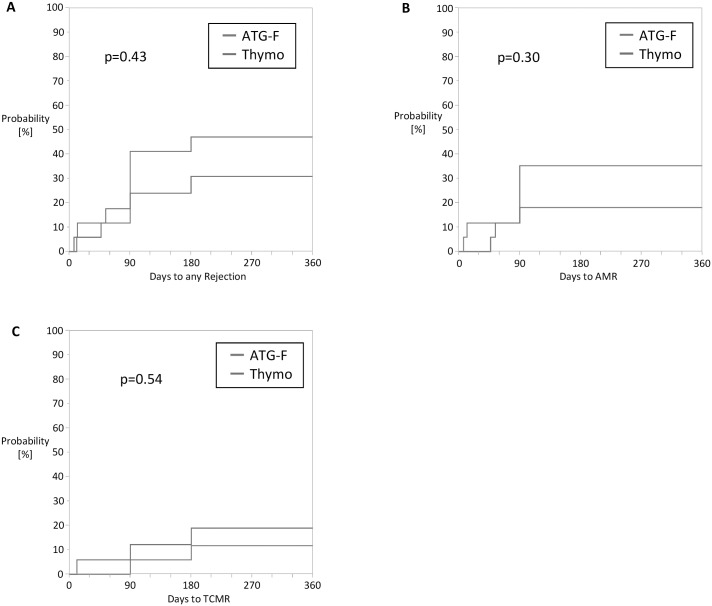
Cumulative incidence of biopsy proven clinical and subclinical rejection episodes in the ATG- and Thymo-Group. (A) Days to any allograft rejection, (B) Days to antibody mediated allograft rejection (AMR), (C) Time to any T-cell mediated allograft rejection (TCMR).

The cumulative incidence of clinical and subclinical antibody mediated allograft rejection (AMR) as well as T-cell mediated allograft rejection (TCMR) was equal in both groups (p = 0.30 and 0.54 respectively) (Fig 4A–4C). There were in total five clinical rejection episodes, two AMR in the ATG-F-group and two AMR and one TCMR in the Thymo-group (p = 0.66). The clinical rejection episodes occurred during the first 44 days after transplantation. The prevalence of subclinical AMR and TCMR at 3 and 6 months post-transplant detected by protocol biopsies were not different between the two groups (p = 0.41 and 0.36 respectively). Clinical and subclinical rejections were treated according to local practice.

There were similar numbers of delayed graft function (DGF) with 4/17 in the ATG-F-group and 6/17 in the Thymo-group (p = 0.71) and one primary non-function in the ATG-F-group. DGF was associated with deceased donors (p = 0.017) with 10/24 (41%) in deceased donors and 0/10 (0%) in living donors, but there was no association of DGF with extended criteria donors (ECD) versus standard deceased donors (p = 0.68). At end of follow-up there were no differences in the median allograft function between the study groups with an estimated GFR (by MDRD formula) of 56 ml/min/1.73m^2^ (range 24–128) in the ATG-F-group and 51 ml/min/1.73m^2^ (range 22–132) in the Thymo-group (p = 0.69) as well as for median proteinuria 12.7 mg/mmol (3–57) and 15.3 mg/mmol (range 1.4–667) respectively (p = 0.66) at 24 months after transplantation (Table 3).

**Table 3:** Outcome data.

	ATG-F (n = 18)	Thymo (n = 17)	p = value
GFR [ml/min/1.73m^2^], median (range)			
month 1	50.7 (29–99)	51.7 (26–86)	0.55
month 6	53.4 (29–119)	49.5 (23–117)	1.00
month 12	52.5 (29–96)	57.1 (39–125)	0.97
month 24	55.9 (24–128)	51.3 (22–132)	0.69
Creatinine [umol/l], median (range)			
month 1	133 (77–225)	127 (87–274)	0.55
month 6	127 (66–211)	140.5 (68–300)	0.89
month 12	129.5 (77–211)	123.5 (64–187)	0.88
month 24	125 (59–252)	134.5 (61–287)	0.60
Delayed graft function, (n/n)%	4/17	6/17	0.71
Primary non function, (n/n)%	1/18	0/17	1.00
Prot/Crea [mg/mmol], median (range)			
month 6	18.2 (6–129)	17.8 (5–346)	0.66
month 12	13.5 (0.06–96)	15.5 (0.06–161)	0.69
month 24	12.7 (3–57)	15.3 (1.4–667)	0.66
1-year patient survival	88.9%	82.4%	0.59
2-year patient survival	88.9%	82.4%	
1-year graft survival	94.4%	100%	0.33
2-year graft survival	94.4%	100%	

## Discussion

This prospective randomised study of ATG-F vs Thymo in high immunological risk kidney recipients with DSA revealed no significant differences regarding safety and efficacy between the study drugs. The absence of drug related reaction during infusion is probably due to the premedication with high dose of intravenous steroids (in total 1000mg Methylprednisolon) several hours before the first infusion of the study drugs, as Kyllonen reported some drug related reaction during infusion of ATG-F using lower premedication dose of steroids (250mg Methylprednisolon) and only shortly before ATG-F infusion [8]. Hematological parameters confirmed the efficacy of lymphocyte-depletion of both drugs with an equally effect on the lymphocyte count after the first dose of both study medications (p = 0.75). Also during follow up there was no difference in the lymphocyte or WBC count between both groups. We observed a sustained low peripheral T- and B-cell count at 6 and 12 months which were similar in both groups. Also Kho et al found a sustained low T- and B-cell count in kidney transplant patients after an induction treatment with a total dose of 6mg/kg/BW of Thymo after one year [9]. They observed dose dependent T-cell recovery as patients with lower doses had T-cell recovery after one year. There are several reports of lower lymphocyte count and later recovery of lymphocyte after induction treatment with Thymo compared to ATG-F [10–12], which we did not observe. There is a known side effect of thrombocytopenia with ATG-F, which is less frequently reported with Thymo [10;13]. Also we observed a significant lower median thrombocyte count at day 1 and day 7 in the ATG-F-group compared to the Thymo-group (p = 0.031 and 0.037 respectively). But the lowest thrombocyte count in the ATG-F-group was 79x10^9^/l and so no patient required thrombocyte substitution or was withheld of further application of ATG-F.

The median hemoglobin at baseline was significant different (p = 0.029) between the study groups with lower hemoglobin in the Thymo-group. Hemoglobin drop at day 1 after transplantation and hemoglobin count at day 1 was equal between the groups. But at day 14 after transplantation hemoglobin was significant lower in the ATG-F-group but thereafter again equal. Also Rostaing L et al observed lower reticulocyte counts in patients after induction treatment with Thymo compared to ATG-F immediately after transplantation and more frequent use of erythropoiesis stimulating agents during the first 14 days posttransplant in these patients [14]. Despite the lower hemoglobin in the ATG-F-group there was not a significant difference in red blood cell transfusion between the two groups.

There was one case of SAE due to postoperative DIC with consecutive fatal outcome with primary allograft failure and death of the patient during follow-up in the ATG-F-group. The risk of DIC in patients receiving ATGs is known but very rare. In patients receiving ATG-F there are only reported cases of DIC with the use of higher dose of ATG-F (10-30mg/kg/BW) in bone marrow transplantation [15]. There was no sign for hyper-acute AMR causing DIC in the histology examination of the removed kidney transplant. So it remains unclear whether ATG-F or another unrecognized trigger was the cause for DIC in this patient.

Infectious complications as known side effect under immunosuppressive therapy are always of concern especially in more intensive immunosuppressive therapies e.g. induction treatment with ATGs. Several studies have demonstrated that viral infections, especially with CMV, but not bacterial or fungal infections, occurred earlier and with a greater incidence in patients receiving induction therapy with Thymo compared to ATG-F [16;17]. Also we found no difference in bacterial or fungal infections between the study groups. But in contrast to these studies we found no significant differences in the incidence of CMV replication or disease between the two groups (p = 0.4). This might be due to the fact that we used VGC prophylaxis in all the patients at risk for CMV-infection and pre-emptive therapy in case of CMV replication before starting or after stopping VGC prophylaxis. Surprisingly, EBV Replication (n = 5) was only observed in the ATG-F-group. All the patients with EBV replication had low level (< 10`000 Geq/ml) EBV-DNA copies by PCR. In the majority of the patients (4/5) it occurred during the first 14 days after transplantation and normalized in all the patients during follow-up. One reason for the only low level transient EBV replication is probably due to the fact that EBV high risk patients (D+/R-) were excluded from the study. Another reason might be that CMV-prophylaxis with VGC had an impact on the rapid normalization of EBV-replication in these patients. It was shown by Höcker et al that VGC in EBV high risk patients (D+/R-) is reducing the incidence of EBV primary infection and in case of primary infection patients with VGC have significant lower EBV viral load [18]. Early EBV reactivation after ATGs is widely described in patients after induction treatment for bone marrow transplantation [19;20]. Mensen et al found a similar incidence of EBV-reactivation in patients who received ATG-F or Thymo for induction treatment for allogenic bone marrow transplantation but significant higher copies in patients who received Thymo. They observed as well a higher incidence of PTLD after Thymo induction compared to ATG-F. Also in kidney transplantation the incidence of PTLD after induction treatment with Thymo seems to be more frequent than with ATG-F [16;21]. Despite the early yet transient EBV replication in some of our patients we observed not a single PTLD during the 24 months follow-up in our study. So the impact of this early EBV replication after ATG-F treatment remains speculative but had no relevant clinical consequence.

Despite induction therapy and triple maintenance immunosuppression the incidence of allograft rejection is still higher in immunological high-risk patients with HLA DSA compared to patients without DSA. Several studies have shown that the incidence of allograft rejection in these patients can be lowered with an induction treatment with ATGs +/- IVIG with an incidence of about 11–15% of clinical rejections [4;22]. Also we observed an overall low incidence of clinical allograft rejection (AMR and TCMR) in only 15% of our patients during the first 24 months after transplantation. However in another 24% of patients we observed subclinical (AMR and TCMR) rejection by protocol biopsies. The cumulative incidence of clinical and subclinical AMR as well as TCMR was not different between the study groups (47% vs 31%; p = 0.43) (Fig 4A–4C). The five clinical rejection episodes occurred during the first 44 days after transplantation and were all successfully treated. There were two patients which were retreated with a second course of ATGs, one patient in combination with rituximab and plasma exchanges. One patient received as well rituximab in combination with plasma exchanges and steroid pulses. The remaining two patients were only treated with steroids pulses.

Ejaz et al showed that ATGs are the backbone of induction treatment in HLA sensitized kidney transplant recipients as even the addition of the B cell targeting agents rituximab and bortezomib alone or in combination had no influence on the overall allograft rejection episodes in these patients compared to ATG alone [23]. These data supporting several observations that ATGs have not only a T-cell depletion effect but as well a strong effect on B-cells [24;25]. Therefore additional B cell targeting agents have no additional benefit. Popow et al found a clear effect on B-cells despite the lack of significant amount of B-cell-specific antibodies with both compounds [7]. In addition, they observed an immunomodulatory effect of ATGs by upregulation of FOXP3 expression and other regulatory T-cells, which was also observed by others [26;27]. Another immunosuppressive effect of both compounds is the induction of apoptosis of naïve, activated B-cells and bone marrow resident plasma cells [28]. Also we observed a sustained low B-cell count at several time points. Krepsova et al found beside a long lasting suppression of T-cells as well suppression of NK-cells after induction treatment with Thymo [29]. All these mechanisms of action together (T-cell depletion, immunomodulatory effects, interference with B cells, NK cells and regulatory T-cells) are providing the potent immunosuppressive effect of ATGs.

Despite the observed cumulative incidence of clinical and subclinical rejection rate of 47% and 31% respectively, the outcome of allograft function at 2 years was very good and similar for both compounds with an estimated GFR of 56 ml/min/1.73m^2^ and in the ATG-F-group and 51 ml/min/1.73m^2^ (p = 0.69) in the Thymo-group. These good results in immunological high-risk patients confirm the efficacy of both study drugs as induction treatment.

Our study has several limitations. The main limitation is the low number of patients included. When using our data with the overall rejection rate of 47% in the ATG-F group and 31% in the Thymo group, we retrospectively calculated a needed total sample size of 142 patients with a statistical power of 80% to detect a significant difference between the two groups. Only a multicentre or nationwide study would have achieved such a high number of patients and so was not feasible in our two centers study. Another limitation is the short-term follow-up. It is known that lymphocyte alteration after induction therapy with ATGs is lasting for more two years [9;30]. So it might be possible that infections and allograft rejection occur with different incidence in the long-term. The same holds true regarding the incidence of PTLD. It was shown by Opelz that PTLD 3-years-incidence was higher with Thymo compared to ATG-F in kidney transplant patients with a continuously rise during the follow-up [21].

In conclusion this unique prospective randomized study found no clinical relevant differences between the ATG-F and Thymo as induction treatment in immunological high-risk patients regarding safety parameters. To combine ATGs compound to IVIG is efficient in a high immunological risk situation where transplant occurs against DSA and allows for a good allograft function at 2 years after transplantation.

## Supporting Information

S1 File Study protocol. (PDF)

S2 File Consort checklist. (PDF)

S1 Table Patient raw data. (PDF)
