# Supplementary material for: A Comparison of Two Types of Rabbit Antithymocyte Globulin Induction Therapy in Immunological High-Risk Kidney Recipients: A Prospective Randomized Control Study
Source: PLoS One. 2016 Nov 17;11(11):e0165233. doi: 10.1371/journal.pone.0165233 (PMC5113896; doi:10.1371/journal.pone.0165233)
Supplement: S1 File — (PDF) [file pone.0165233.s001.pdf]

PROTOCOL

An open, multicenter, randomised, parallel group pilot study to investigate two different polyclonal rabbit immunoglobulin preparations for safety and efficacy:

A comparison of ATG-Fresenius S to Thymoglobulin in prophylaxis for immunological high risk patients following renal transplantation

---

PRINCIPAL INVESTIGATOR:	Prof. Jürg Steiger<br>Basel, Switzerland
PHASE OF CLINICAL TRIAL:	IV (Pilot)
INVESTIGATIONAL DRUGS:	ATG-Fresenius S /<br>Thymoglobulin
STUDY-NUMBER:	A-T-Study
STATUS OF THE PROTOCOL:	19.11.2008

PRINCIPAL INVESTIGATOR:

Prof. Dr. J. Steiger
Div. of Transplantation
Immunology and Nephrology
Department of Internal Medicine
University Hospital Basel
Petersgraben 4
CH-4031 Basel
Phone: +41-61-265 44 04

____
Date, Signature

CO-INVESTIGATORS:

PD Dr. M. Weber
Department of Surgery
Visceral and Transplantation Surgery
University Hospital Zürich
Rämistrasse 100
CH-8091 Zürich
Phone: +41-1-255 38 68

____
Date, Signature

PD Dr. M. Dickenmann
Div. of Transplantation
Immunology and Nephrology
Department of Internal Medicine
University Hospital Basel
Petersgraben 4
CH-4031 Basel
Phone: +41-61-265 44 39

____
Date, Signature

Dr. S. Schaub
Div. of Transplantation
Immunology and Nephrology
Department of Internal Medicine
University Hospital Basel
Petersgraben 4
CH-4031 Basel
Phone: +41-61-265 45 33

____
Date, Signature

TABLE OF CONTENTS **Page**

**1.**	**GENERAL INFORMATIONS**	**6**
**2.**	**BACKGROUND INFORMATIONS**	**8**
2.1	Name and description of the investigational product (ATG-Fresenius S)	8
2.2	Summary of nonclinical and clinical trials (ATG-Fresenius S)	8
2.2.1	Pharmacology	8
2.2.2	Toxicology	8
2.2.3	Pharmacokinetics	9
2.2.4	Clinical trials	9
2.3	Name and description of the investigational product (Thymoglobulin)	13
2.4	Summary of nonclinical and clinical trials (Thymoglobulin)	13
2.4.1	Pharmacology	13
2.4.2	Pharmacokinetics	13
2.4.3	Clinical trials	14
2.5	Known and potential risks and benefits to human subjects	14
2.5.1	Risks (ATG-Fresenius S)	14
2.5.2	Benefits (ATG-Fresenius S)	15
2.5.3	Risks (Thymoglobulin)	16
2.5.4	Benefits (Thymoglobulin)	17
2.6	Confirmation	17
**3.**	**TRIAL OBJECTIVES AND PURPOSE**	**18**
**4.**	**TRIAL DESIGN**	**20**
4.1	Type of study, description of study design	20
4.2	Randomisation	20
4.3	Primary and secondary endpoints	21
4.3.1	Primary endpoints	21
4.3.2	Secondary endpoints	22
4.4	Time schedule	22
4.5	Discontinuation criteria	22
4.6	Drug responsibility	23
**5.**	**SELECTION AND WITHDRAWAL OF SUBJECTS**	**25**
5.1	Study population	25
5.2	Inclusion criteria	25
5.3	Exclusion criteria	25

6. TREATMENT OF SUBJECTS 26
6.1 Administration route, single dose, dosage, treatment period, follow-up for subjects 26
6.2 Transplant monitoring 26
6.3 Diagnosis and treatment of rejection episodes 27
6.4 Prophylaxis and treatment of infections 27
6.5 Procedures for monitoring subject compliance 30
6.6 Handling of the investigational drug 31

7. ASSESSMENT OF SAFETY 32
7.1 Specification of safety parameters 32
7.2 Methods and timing for assessing, recording, and analysing of safety parameters 32
7.3 Procedures for eliciting reports of and for recording and reporting adverse events and intercurrent illnesses 32
7.4 Type and duration of the follow-up for subjects after adverse events 35
7.5 Report of serious adverse events 35

8. ASSESSMENT OF EFFICACY 36

9. STATISTICS 36

10. ETHICS AND LEGAL REQUIREMENTS 37
10.1 Description of ethical considerations relating to the trial 37
10.2 Patient Informed Consent 37
10.3 Notification 38

11. DATA HANDLING AND RECORD KEEPING 38
11.1 Direct access to source data/source documents 38
11.2 Identification of source data 38
11.3 Registration of data 38
11.4 Record of the termination of the study 39
11.5 Record keeping 39
11.6 Monitoring 39
11.7 Publication 39

12. INSURANCE 40

13. LITERATURE 41

14. ENCLOSURES 44

1. GENERAL INFORMATIONS

Title of the protocol:
An open, multicenter, randomized, parallel group pilot study to investigate two different polyclonal rabbit immunoglobulin preparations for safety and efficacy: A comparison of ATG-Fresenius S to Thymoglobulin in prophylaxis for immunological high risk patients following renal transplantation

Protocol identification number: A-T-Study
Date: 28.8. 2008

Amendment number: 1
Date: 28.8.08

Name/Address/Phone of Principal Investigator:
Prof. Dr. J. Steiger
Div. of Transplantation
Immunology and Nephrology
Department of Internal Medicine
University Hospital Basel
Petersgraben 4
CH-4031 Basel
Phone: +41-61-265 44 04

Responsible person for all trial-site related medical decisions:
PD Dr. M. Weber
Department of Surgery
Visceral and Transplantation Surgery
University Hospital Zürich
Rämistrasse 100
CH-8091 Zürich
Phone: +41-1-255 38 68

PD Dr. M. Dickenmann
Div. of Transplantation
Immunology and Nephrology
Department of Internal Medicine
University Hospital Basel
Petersgraben 4
CH-4031 Basel
Phone: +41 61-265 44 39

Dr. S. Schaub
Div. of Transplantation
Immunology and Nephrology
Department of Internal Medicine
University Hospital Basel
Petersgraben 4
CH-4031 Basel
Phone: +41-61-265 45 33

Name/Address of laboratories etc.:

1. University Hospital Basel
   Petersgraben 4
   CH-4031 Basel

2. University Hospital Zürich
   Rämistrasse 100
   CH-8000 Zürich

2. BACKGROUND INFORMATION

2.1 Name and description of the investigational product (ATG-Fresenius S)

ATG-Fresenius S consists of highly purified immunoglobulin, derived from rabbits after immunisation with a T-lymphoblast line (Jurkat cell line). This immunoglobulin (IgG fraction) is dissolved in sterile, apyrogenic, hypotonic, acidic solution. The final product is a concentrate formulated as 5 ml of sterile solution in 8 ml glass vials. It contains neither stabilizers nor preservatives. Each ml of ATG-Fresenius S contains:

- 20 mg rabbit immunoglobulin
- $NaH_2PO_4 \times H_2O$ (10 mM)
- phosphoric acid (85% q.s.) ad pH 3.4 - 4.0
- water ad 1 ml.

2.2 Summary of nonclinical and clinical trials (ATG-Fresenius S)

2.2.1 Pharmacology

ATG-Fresenius S as a polyclonal anti-T-cell antibody solution is described to have a direct effect on T-cells, thus resulting in a T-cell depletion. The main mechanism of action of T-cell depletion is opsonisation and lysis following complement activation.

Published results of *in-vivo* and *in-vitro* tests indicate that the effect of ATG-Fresenius S is among other effects due to binding to
$CD1a$, $CD2^+$, $CD3^+$, $CD4^+$, $CD5^+$, $CD7^+$, $CD8^+$, $CD21^+$, $CD28^+$, CD45(RA), $CD54^+$, $CD71^+$, $CD80^+$, $CD86^+$, $CD95^+$, α/β-TCR, CTLA-4, $LFA\text{-}2^+$ and $ICAM\text{-}1^+$ lymphocytes.
With these receptors mainly T-lymphocytes are characterised. CD5 is also expressed on the surface of proliferating B-lymphocytes.

Spontaneous rosette formation of human T-lymphocytes in the presence of sheep erythrocytes is inhibited by ATG-Fresenius S.

The survival of allogeneic skin grafts in the rhesus monkey is increased following administration of ATG-Fresenius S.

ATG-Fresenius S is virtually free of possible cross-reacting antibodies reacting with erythrocytes, human plasma proteins and glomerular basement membranes.

2.2.2 Toxicology

The tests on acute toxicity were carried out in the rabbit and the rhesus monkey.

Even with intravenous administration of 900 mg/kg bw, the rabbits demonstrated no pathological changes in either the clinical signs or in the hematological test results.

With a dosage of 100 mg/kg bw in the rhesus monkey, only in the first 3 days a slight motory inhibition, a shift in the neutrophilic granulocytes in the hemogram,

and a temporary decrease of the reticulocytes and thrombocytes were observed.

The determination of the sub-acute (chronic) toxicity was performed in the rhesus monkey. The intravenous application of 300 and 500 mg/kg bw/day led on the 7th day (300 mg) and on the 5th day (500 mg) to the death of the experimental animals. The toxic symptoms indicate an anaphylactic shock with circulatory collapse as the cause of death.

In comparison to the control group, there was a decreasing lymphocyte count in all dosage groups. The histological findings and the other hematological findings lay within the normal range. An activation of the lymphatic organs could not be determined in any of the experimental animals.

An influence on the central nervous system (CNS) by the administration of ATG-Fresenius S can be excluded by the results of the trials in the conscious cat. The trials in the anaesthetized cat gave no indications of cardiovascular side effects.

Furthermore, ATG-Fresenius S had no mutagenic effect in 3 different *in-vitro*-tests, both with and without metabolic activation.

2.2.3 Pharmacokinetics

ATG-Fresenius S is administered through the intravenous route and therefore is 100% bioavailable.

ATG-Fresenius S is subject to protein metabolism as are other bodily proteins. No unphysiological metabolites are known to exist. The half-time of ATG-Fresenius S is approximately 14 days (in case of a dosage of 4 mg/kg bw/d over 7 days).

2.2.4 Clinical trials

Besides a multitude of published investigations following comparative clinical trials were initialised to compare the two investigational products:

1. NORRBY and OLAUSSON (1997) (11) summarized 90 patients in a randomized short-term trial using ATG-Fresenius S or Thymoglobulin. Main focus was on the safety aspects, infectious complications, rejection frequency, and patient and graft survival.

   As induction therapy one group received 5 mg/kg/d ATG-Fresenius S the other group was treated with 2.5 mg/kg/d Thymoglobulin; both treatments were administered over a period of 4 to 7 days.

   During the follow-up of 6 months rejection episodes occurred in 67% in the Thymoglobulin group and in the ATG-Fresenius S group in 58%, which was not significantly different. The blood profile showed, however, significant differences. Thymoglobulin lowered the white blood cell count more than the ATG-Fresenius S at day 3 and 7. This change in the white blood cell count was even more pronounced when compared to the pre-operative value for each patient.

   Regarding adverse events the Thymoglobulin group suffered a little bit more of fever but no differences were seen in life-threatening complications

although the scores of arrhythmia, blood pressure decrease, and respiratory symptoms were slightly higher in the Thymoglobulin group.

No difference was noted regarding graft and patient survival and infectious complications. Although there was no statistical significance concerning CMV infection, the Thymoglobulin group had a higher infection rate (40%) than the ATG-Fresenius S group (22.2%).

2. In the same year THERVET et al. (1997) (22) published an *in vitro* study to monitor T-cells during Thymoglobulin induction therapy to compare the efficacy and the adverse events observed with different Thymoglobulin regimens and to correlate the T-cell subset monitoring with the different clinical events.

   They divided a total of 31 patients into three groups. The first group of 12 patients received 75 mg/d, the second group of 12 patients was treated with 1 mg/kg bw/d, and the third group of 8 patients was treated with a variable dose. The inclusion criteria was a kidney transplantation, sex and gender were not specified.

   The criteria for the evaluation was the analysis of CD2+, CD3+, CD4+ and CD8+ T-cells, absolute lymphocyte count, T-cell rosetting, patient/graft survival, creatinine, and CMV-infection.

   THERVET et al. discovered that the patient and graft survival, incidence of acute rejection or CMV infection, and serum creatinine were not different between the groups. In all groups, the number of total lymphocytes, E+, CD2+, CD3+, CD4+, and CD8+ T-cells were severely suppressed rapidly after Thymoglobulin administration. The recovery was significantly longer when the higher dosage of Thymoglobulin was used. A positive correlation between the lymphocyte count before transplantation, but not during the phase of induction therapy, and the occurrence of acute rejection was found. The T-cell subset analysis shows no correlation with the other outcome. They also did not see a difference in the CMV-infection rate.

3. In the beginning of 2000 DJAMALI et al. (3) published their experience with two different regimen of Thymoglobulin: one group of 23 patients received a fixed daily dose of 50 mg Thymoglobulin and the other an intermittent dose (50 mg for the first 3 days and then intermittently only if CD3+-lymphocytes were >10/mm^3. Both groups had the same basic immunosuppression consisting of steroids, azathioprine and cyclosporin. There were no significant differences in acute rejection rates (13% vs. 19%) or infection rates (CMV: 35% vs. 19%; bacterial: 34% vs. 31%). Since a similar T-cell depletion was achieved in both groups the authors propose this intermittent approach as an induction protocol, particularly in patients with poor graft function in whom cyclosporin introduction has to be delayed or those with increased risk of CMV infections or secondary malignancies.

4. A retrospective, single-center study evaluated the influence of ATG-Fresenius S vs. Thymoglobulin on the occurrence of different long-term clinical effects (DUCLOUX et al. 2004) (4). 194 patients underwent a renal transplantation between June 1993 and April 2001. 129 patients were treated with ATG-Fresenius S and a total of 65 received Thymoglobulin.

The induction therapy consisted of the prolonged bolus regime for the ATG-Fresenius S group (day 0: 9mg/kg; day 1-4: 3mg/kg/d). The treatment of the Thymoglobulin group was adapted within the study period (day 0: 5mg/kg; days 1-4: 2mg/kg/d until May 1996; day 0: 3mg/kg; days 1-4: 1mg/kg/d from June 1996 – May 1998); day 0: 2mg/kg; days 1-4: 1mg/kg/d from June 1998 on).

The main result of this study was that the Thymoglobulin treatment caused more CMV diseases (37%, p=0.02) and post-transplant malignancies (12.3%, p=0.01) compared to ATG-Fresenius S (CMV=23%, malignancy=3.9%), without any significant beneficial effect on the rate of acute rejection (p=0.19). The investigators found that this difference was mainly caused by an increased incidence of lymphoma in the Thymoglobulin group (Thymoglobulin=4.6% vs. ATG=1.5%; p=0.1).

Another important result was the lower patient survival rate in the Thymoglobulin group (ATG=96.1% vs. Thymoglobulin=86.2%, p=0.005). The patients treated with Thymoglobulin showed a fourfold increased risk of death compared with patients who were treated with ATG-Fresenius S. This increased risk was even greater when only immunosuppression-related deaths were considered. The conclusion was that Thymoglobulin is a more aggressive immunosuppressive agent than ATG-Fresenius S and carries a significantly increased risk of viral and neoplastic complications (p=0.02 and p=0.01).

It was recommended that this retrospective study should be confirmed by a prospective randomised trial with a long-term follow-up. *Referring to this recommendation this trail will be performed.*

Beside these comparative studies following investigations were performed due to the desire to reduce the costs for transplantations.

5. To safe money KADEN et al. (1992) (8) published their experience with a single bolus ATG-Fresenius S therapy in non-sensitised patients. In this retrospective analysis 204 patients were studied exactly half of whom had a panel-reactive-antibody (PRA)-titer lower than 5% and half of whom had either PRA higher than 5% or a second or higher repeat renal graft. Both PRA-determinated groups were subdivided into those with or without ATG-Fresenius S added to their basic treatment (triple drug therapy (TDT) consisting of CsA, Aza, and P). In the low-PRA-titer/ATG-Fresenius S group only one intra-operative bolus of 9 mg/kg bw was administered, while in the high-PRA-titer/ATG-Fresenius S group the immunoglobulin was continued at 1.5 - 3 mg/kg bw/d (T-cell count targeted below 200/µl) over 8 days after the bolus of 3 mg/kg bw intra-operatively. In the ATG-Fresenius S groups the incidence of rejection was reduced by approximately 50%. Moreover, in the high-PRA-titer group, the graft survival rate after 12 months was higher (82% vs. 71%) in the ATG-Fresenius S group compared with the patients not receiving ATG-Fresenius S. Although CMV-seropositivity was significantly increased in the ATG-Fresenius S groups, severe CMV-infections were not elevated nor was there a higher incidence of other major infections in these groups. Even in the non-sensitised group, there was a 6% difference in one-year graft survival in favor of ATG-Fresenius S (92%) vs. control (86%).

6. In 1995 KADEN et al. (9) confirmed their results in a second publication relating to 356 patients (254 patients treated with TDT + ATG-Fresenius S bolus and 102 patients treated with TDT alone). The impressive outcome, after 2-year follow-up, was: graft survival rate 91% vs. 74%; patient survival rate 96% vs. 90% for the TDT + ATG-Fresenius and TDT groups, respectively. The results clearly indicated the benefit of including ATG-Fresenius S bolus in an induction treatment protocol. In their recent publication, KADEN et al. (1997) (10) reported the results of the 3-year follow-up of this patient population. The results were: graft survival rate 87.2% vs. 71.6%; patient survival rate 94.9% vs. 87.2% for the TDT + ATG-Fresenius S group and the TDT group, respectively. The authors concluded that the longer the follow-up time, the more significant were the differences in survival.

7. SAINT-HILLIER et al. (1994) (14) confirmed the observations of KADEN et al. (1992) (8). A bolus of 9 mg/kg bw ATG-Fresenius S within 3 hours pre- or intra-operatively was administered in 31 patients and in 6 patients, who were hyperimmunised or re-transplanted, and continued with ATG-Fresenius S at a dose of 3 mg/kg bw/d over 4 days. CsA was introduced at day 5 or earlier if S-Crea decreased by 30% of the pre-transplant level. It was found that only 22% of the kidneys were rejected and one patient with Wegener's disease lost his graft. The average S-Crea level of the patients was 158 µmol/l at the most recent follow-up.

8. Due to this experience, SAINT-HILLIER et al. (1997) (15) expanded this therapeutic approach to 17 patients at high immunological risk (recipients of second and third transplants). ATG-Fresenius S 9 mg/kg bw was administered pre- and intra-operatively, followed by 3 mg/kg bw/d for 4 days combined with steroids, AZA and low dose CsA. After a mean of 30 months of follow-up actual patient and graft survival rates were 100%. Only 5 patients (29%) had acute reversible rejections, which responded to a single pulse of MP. Interestingly, a sharp increase of IL-10 levels was found after the first infusion of ATG-Fresenius S. The levels rose from 15 - 28 pg/ml to 660 $\pm$ 789 pg/ml and returned after 24 h to normal levels. IL-2 and IFN-γ were inhibited simultaneously. The massive release of IL-10 was assumed to contribute to graft tolerance.

9. In a further approach SAINT-HILLIER et al. (16) compared bolus therapies with ATG-Fresenius S (n=56) and Thymoglobulin (n=37) in 2000. The ATG-Fresenius S regimen was the same as described above. For Thymoglobulin they used a pre-transplant bolus of 4 mg/kg and after transplantation 2 mg/kg/d for 4 days with the same triple drug therapy. After a follow-up of 3 years the acute rejection rate was twice as high in the Thymoglobulin group (12.5% vs. 24%), patient and graft survival was 98% vs. 77% and 96% vs. 77% for ATG-Fresenius S and Thymoglobulin, respectively.

2.3 Name and description of the investigational product (Thymoglobulin)

Thymoglobulin is an immunosuppressive polyclonal rabbit anti-human thymocyte immunoglobulin. One vial of Thymoglobulin contains 25 mg of purified lyophilised immunoglobulin which must be reconstituted using a 5 ml vial of diluent which is added additionally. The vial of the freeze-dried product contains:

- Rabbit anti-human thymocyte immunoglobulin
- 50 mg Glycine
- 10 mg Sodium chloride
- 50 mg Mannitol to ensure stability

These additives regulate the pH, stability and isotonicity.

Diluent:

- sterile water for injection.

The reconstituted solution has a pH of 7.0 ± 0.4 and contains no preservatives.

2.4 Summary of non clinical and clinical trials (Thymoglobulin)

2.4.1 Pharmacology

Thymoglobulin is a polyclonal anti-thymocyte antibody injectable solution of purified immunoglobulin obtained by immunization of rabbits with human thymocytes. Human red blood cells are used in the manufacturing process to deplete cross-reactive antibodies to non-T-cell antigens. The manufacturing process is validated to remove or indicate potential exogenous viruses. Possible mechanisms by which Thymoglobulin may induce immuno-suppression *in vivo* include: T-cell clearance from circulation and modulation of T-cell activation, homing, and cytotoxic activities. Thymoglobulin includes antibodies against T-cell markers such as CD2, CD3, CD4, CD8, CD11a, CD18, CD25, CD44, CD45, HLA-DR, HLA Class I heavy chains, and β2 microglobulin.
In vitro Thymoglobulin mediates T-cell suppressive effects via inhibition of proliferative responses to several mitogens. Thymoglobulin has not been shown to be effective for treating antibody (humoral) mediated rejections. Each Thymoglobulin lot is released following potency testing (lymphocytotoxicity and E-rosette inhibition assays), and cross-reactive antibody testing (hemag-glutination, platelet agglutination, anti-human serum protein antibody, antiglomerular basement membrane antibody, and fibroblast toxicity assays) on every lot.

2.4.2 Pharmacokinetics

After an intravenous dose of 1.25 to 1.5 mg/kg/d (over 4 hours for 7-11 days) 4-8 hours post-infusion, Thymoglobulin levels were on average 21.5 µg/ml (10 – 40 µg/ml) with a half-life of 2-3 days after the first dose, and 87 µg/ml (23-170 µg/ml) after the last dose.

2.4.3 Clinical trials

A controlled, double-blind, multicenter, randomized clinical trial by GABER et al. (1) comparing Thymoglobulin and ATGAM was conducted at 28 US transplant centers in renal transplant patients (n=163) with biopsy-proven Banff Grade II (moderate), Grade III (severe), or steroid-resistant Grade I (mild) acute graft rejection. This clinical trial rejected the null hypothesis that Thymoglobulin was more than 20% less effective in reversing acute rejection than ATGAM. The overall weighted estimate of the treatment difference (Thymoglobulin-ATGAM success rate) was 11.1% with a lower 95% confidence bound of 0.07% (27).

In the study, patients were randomized to receive 7 to 14 days of Thymoglobulin (1.5 mg/kg/d). For the entire study, the two treatment groups were comparable with respect to donor and recipient characteristics.

There were no significant differences between the two treatments with respect to day 30 serum creatinine levels relative to baseline, improvement rate in post-treatment histology, one-year post-rejection Kaplan-Meier patient survival (Thymoglobulin 93%, n=82 and ATGAM 96%, n=80), day 30 and one-year post rejection graft survival (Thymoglobulin 83%, n=82; ATGAM 75%, n=80).

Thymoglobulin adverse events were generally manageable or reversible. In the US Phase III controlled clinical trial (n=163) comparing the efficacy and safety of Thymoglobulin and ATGAM, there were no significant differences in clinically relevant adverse events between the two treatment groups. Malignancies were reported in 3 patients who received Thymoglobulin and in 3 patients who received ATGAM during the one-year follow-up period.

2.5 Known and potential risks and benefits to human subjects

2.5.1 Risks (ATG-Fresenius S)

Contraindications:
ATG-Fresenius S must not be administered

- in the presence of allergy to rabbit proteins
- in patients with severe thrombocytopenia, i.e. less than 50,000 platelets/µl (as ATG-Fresenius S may decrease the number of thrombocytes and thus increase the risk of haemorrhagia)
- in patients with bacterial, viral, or mycotic infections, which are not under therapeutic control
- if pregnancy is suspected or verified. (In case of pregnancy, there are neither clinical experiences nor experiences in animals available. Thus the therapy with ATG-Fresenius S is under the risk/benefit evaluation of the physician. It should be considered, that at least human IgG can pass the placental barrier.)

Side effects:
At any time during the application of ATG-Fresenius S or shortly after the in-fusion there is a risk of anaphylactic reactions including drop in blood pressure, chest pain, fever, or urticaria. These symptoms usually are seen only during the first application of ATG-Fresenius S and do not appear under later applications.

In case the symptoms become clinically significant, stop the infusion and immediately begin anaphylaxis and shock management.

As with other heterologous antisera, serum sickness may develop after 8 - 14 days of ATG-Fresenius S therapy. However, ATG-Fresenius S therapy needs not to be suspended, if the symptoms remain mild and reversible.

The pronounced decrease of lymphocytes, particularly T-lymphocytes is preparation-specific and results from the immunosuppressive efficacy of ATG-Fresenius S. Thrombocytopenia and granulocytopenia requiring discontinuation of therapy are rare.

Interactions:
Combination with other immunosuppressive drugs (corticosteroids, azathioprin, cyclosporin A, Cellcept, FK506) will increase the risk of infection, thrombocytopenia, and anaemia. Patients receiving this kind of therapy require careful monitoring. Adaptation of the concomitant immunosuppressive drug is also recommended. For immunosuppressed patients live-attenuated virus vaccination is contraindicated. Other vaccines may result in suboptimal immunisation results.

2.5.2 Benefits (ATG-Fresenius S)

Due to the particular production procedure of ATG-Fresenius S clinical experience demonstrated that the product is a „mild“ and selective immunosuppressive agent compared with other preparations.

Many *in-vitro* analyses showed that the cytotoxic efficacy of ATG-Fresenius S is lower than that of other immunoglobulin preparations, if lymphocytes of healthy volunteers are used. Performing the same test with lymphocytes of patients with renal failure under dialysis, the efficacy of ATG-Fresenius S is increased 10- to 15-fold. A clue to this observation may be, that the immunisation of rabbits with a human lymphoblast cell line results in an antibody pattern specific for activated lymphocytes. In lymphocyte specimens from normal healthy volunteers used in laboratory settings only few lymphocytes are activated. As a consequence, the cytotoxic effect of ATG-Fresenius S may be low compared to other immunoglobulins. With increasing activation of the specimen used, the cytotoxicity of ATG-Fresenius S will increase.

This explanation is also supported by the experience that ATG-Fresenius S therapy does not result in similar low T-cell counts as during therapies using other immunoglobulins.

Thus, ATG-Fresenius S recognizes lymphocytes being activated after contact with the graft, whilst the cytotoxic effect against non-activated, resting or silescent lymphocytes is minimal. Compared with other polyclonal immunoglobulin preparations the efficacy of ATG-Fresenius S is considered to be more specific.

As a consequence of the more specific elimination of lymphocytes those non-activated cells escape destruction, which may have an important impact in the management of infections (T_H-lymphocytes, memory-cells etc.). Because of that, the patient’s own defence mechanisms are maintained. This is emphasized by the comparably low incidences of infections (in particular CMV-infections) after ATG-Fresenius S therapy.

SINHA et al. (1993) (18) published that EBV causes infectious mononucleosis and is related to Burkitt's lymphoma, nasopharyngeal carcinoma, mononucleosis, and B-cell lymphomas in immunodeficiency cases as well as it is associated with cancers of T-cells wherein the T-cell lymphomas are positive for the EBV genome.

It is known that EBV infects cells via the CR2 (CD21) that was previously thought to be expressed only on the surface of B-cells and certain epithelial cells but the latest findings showed that the EBV receptor is also present on T-cells. The study demonstrated that Jurkat human T-cells express a molecule that reacts with both anti-CR2 antibodies and the third component of complement C3. Furthermore the data indicated that this molecule binds EBV detected by incubation with biotin-conjugated virus and streptavidin phycoerythrin.

It was seen that EBV variably infects Jurkat cells as demonstrated by the presence of transcripts of Epstein-Barr nuclear Ag (EBNA-1) using the polymerase chain reaction.

As the Jurkat cell line is used for the immunisation of rabbits it can be concluded that ATG-Fresenius S contains anti-CD21 antibodies interfering with EBV-binding and thus contributing to the relatively low rate of occurrence of EBV-mediated lymphoma (post-transplant lymphoproliferative disease, PTLD) after treatment.

2.5.3 Risks (Thymoglobulin)

Thymoglobulin is indicated for the prophylaxis or treatment of renal transplant acute rejection in conjunction with concomitant immunosuppression. It is contraindicated for in patients with history of allergy or anaphylaxis to rabbit proteins, or who have an acute viral illness.

In a case of anaphylaxis, the infusion should be terminated immediately. Medical personnel should be available to treat patients who experience anaphylaxis. Emergency treatment such as 0.3 ml to 0.5 ml aqueous epinephrine subcutaneously and other resuscitative measures including oxygen, intravenous fluids, antihistamines, corticosteroids, pressor amines, and airway management, as clinically indicated, should be provided.

Drug interactions

- Because Thymoglobulin is administered to patients receiving a standard immunosuppressive regimen, this may predispose patients to over-immunosuppression.
- Thymoglobulin can stimulate the production of antibodies which cross-react with rabbit immunoglobulins.

Thymoglobulin is contraindicated in patients with history of allergy or anaphylaxis to rabbit proteins, or who have an acute viral illness.

Anaphylaxis has been reported with Thymoglobulin use. In such cases, the infusion should be terminated immediately.

Thrombocytopenia or neutropenia may result from cross-reactive antibodies and is reversible following dose adjustments.

To minimize first dose effects premedication with corticosteroids, acetaminophen, and/or an antihistamine is recommended.

Prolonged use or overdosage of Thymoglobulin in association with other immunosuppressive agents may cause over-immunosuppression resulting in severe infections and may increased the incidence of lymphoma or post-transplant lymphoproliferative disease (PTLD) or other malignancies. Appropriate antiviral, antibacterial, antiprotozoal, and/or other antifungal prophylaxis is recommended.

During Thymoglobulin therapy, monitoring the lymphocyte count may help to assess the degree of T-cell depletion. For safety, WBC and platelet counts should also be monitored.

The carcinogenic and mutagenic potential of Thymoglobulin and its potential to impair fertility have not been studied.

Animal reproduction studies have not been conducted with Thymoglobulin. It is also not known whether Thymoglobulin can cause fetal harm or can affect reproduction capacity. Thymoglobulin should be given to a pregnant woman only if clearly needed.

2.5.4 Benefits (Thymoglobulin)

Thymoglobulin is a polyclonal anti-thymocyte globulin, obtained by immunization of rabbits with human thymocytes. Thymoglobulin contains cytotoxic antibodies directed against antigens expressed on human T-lymphocytes. After transplantation these are mostly T-lymphocytes that recognize the foreign antigens carried by the graft.

Immunosuppressive treatment with Thymoglobulin for 7-14 days following renal transplantation decreases the incidence of acute rejection compared to conventional triple drug therapy. This reduction in rejection rate is not associated with an increase in side effects due to (over)immunosuppression.

2.6 Confirmation

With the signature on page „Responsibilities“ the responsible persons for this clinical trial confirm to conduct the trial in compliance with the protocol, ICH-GCP, the applicable regulatory requirements, and the Declaration of Helsinki (enclosure 1).

3. TRIAL OBJECTIVES AND PURPOSE

Reflecting the last decades of clinical organ transplantation the accompanying immunosuppressive therapy developed from single and double to triple, quadruple, or sequential strategies using high-dose or low-dose regimens (6, 12, 13, 19, 21).

Recent multi-drug immunosuppressive protocols attempt to combine the advantage of different mechanisms of action by blocking signal or activation pathways of immunocompetent cells with a dose reduction of each single drug to decrease side effects (5).

Despite all these advantages rejections of allografts - especially steroid resistant rejections (SRR) - do occur and remain one of the major reasons for graft loss and patient death. Undoubtedly one of the most powerful weapons to treat rejection episodes are anti-lymphocytotoxic immunoglobulin preparations (7, 17), a treatment that is generally accepted worldwide for this indication.

The use of immunoglobulins (Ig) in prophylaxis is still in ongoing controversial discussion. The benefit of reducing the incidence of rejections, early graft loss, and dialysis after transplantation, in patients receiving Ig-induction therapy (2) faces arguments regarding necessity of general Ig-induction therapy, the waste of efficacious drugs for the management of SRR, higher rate of infections, and the costs of the treatment (23).

Considering only high-risk patients (i.e., patients who lost their graft due to immunological reasons or patients with high levels of panel reactive antibodies) an Ig-induction therapy is widely accepted, setting the costs of the therapy in relation to the improved outcome of these patients.

Nevertheless, it is desirable to reduce the costs furthermore. A possible way to save money might be the strategy of KADEN et al., who published his experience with a single bolus ATG-Fresenius S therapy in non-sensitised patients 1992 (8).

The bolus ATG protocol cannot, however, be adapted without further investigation, since another report (24) mentions a relatively high incidence of graft vascular thrombosis in patients who received a single high dose of ATG from another manufacturer.

This fact and the results from the references in chapter 2 formed the idea to investigate a study to obtain preliminary information on the safety and efficacy of two induction treatment protocols – ATG-Fresenius S as a bolus regimen and Thymoglobulin in standard application – in immunological risk patients under basal immunosuppression with Tacrolimus, MMF and steroids used in the prevention of acute rejection following renal transplantation.

All AE will be documented but not all AE’s will be classified as primary endpoint.

The trial will be performed as a pilot trial to compare tolerance, side effects and efficacy of ATG-Fresenius S compared to Thymoglobulin.

Clinical equivalence of ATG-Fresenius S to Thymoglobulin will be assumed up to a difference rejection in rates of ± 15% absolute.

Two immunosuppressive protocols will be compared:

1. a regimen consisting of a triple drug therapy with Tacrolimus, MMF, and steroids as basis therapy plus IVIG for 5 days (total dose 2g/kg bw, max. 140g). In addition, the patients receive intra-operative 9 mg/kg bw ATG-Fresenius S (completed before reperfusion), followed by 3 mg/kg bw/d for the next 4 consecutive days.

vs.

2. a regimen consisting of a triple drug therapy with Tacrolimus, MMF, and steroids as basis therapy plus IVIG for 5 days (total dose 2g/kg bw, max. 140g). In addition, the patients receive intra-operative 1.5 mg/kg bw Thymoglobulin (completed before reperfusion), followed by 1.5 mg/kg bw/d for the next 3 consecutive days.

After kidney transplantation in end stage renal disease patients it is assumed that:

- the safety profile of ATG-Fresenius S may be superior to that of Thymoglobulin
- the efficacy profile of both treatments is clinically equivalent

The aim is to obtain preliminary information on the safety and efficacy of two induction treatment protocols – ATG-Fresenius S and Thymoglobulin – in immunological high risk patients under basal immunosuppression with Tacrolimus, MMF and steroids used in the prevention of acute rejection following renal transplantation.

4. TRIAL DESIGN

4.1 Type of study, description of study design

The trial is planned as a multicenter, 1:1 randomised, parallel group, comparative, open study. The study may be expanded to other Swiss or a foreign state centers. The patients included into the study will receive standard immunosuppression therapy consisting of Tacrolimus / MMF / steroids / IVIG / ATG-Fresenius S bolus therapy in one arm and in the other arm Tacrolimus / MMF / steroids / IVIG / Thymoglobulin, respectively.

4.2 Randomisation

To avoid bias due to patient selection the patients will be assigned to the investigational treatments by randomization. Patients will be assigned a consecutive number in the order in which they enter the trial. The assignment of consecutive numbers will be done within each participating center. Each center will receive a set of sealed random code envelopes which will be labelled with an identification of the trial and a unique treatment number and will contain a note with the identity of treatment assigned to this number. After a patient has been included into the trial, his/her eligibility has been confirmed and he/she is ready to receive the investigational treatment, the investigator will break the seal of the envelope bearing the lowest available number and will administer the treatment corresponding to the included note. The random code envelope must not be opened before the eligibility of a patient has been finally determined and he/she is ready for treatment. Details will be given to the participating investigators.

Patient population:
Recipients, who are at least 18 years or older and have a high immunological risk defined by:
The presence of at least one donor-specific antibody (class I and/or II) detected and specified by flow-technology (FlowPRA and single antigen Flow-beads), which are

- For class I below the threshold of detection of a current CDC T-cell-/ and B-cell cross-match
- For class II below the threshold of detection of a current CDC B-cell cross-match

Screening failure (Drop-out)
Patients can be replaced by new patients before being randomised. After the randomisation has been performed a replacement of a drop-out is not allowed. Every patient that has been randomised and included in the study will be considered at least in the safety analysis.

To ensure an unbiased assessment of AEs a blind cross-over evaluation of all events will be done. The physicians in Basel will assess the causal relationship of AEs to the investigational treatment in patients treated in Zürich and *vice versa*. The appraisal will be done provided by the treatment e.g. either with ATG-Fresenius S or Thymoglobulin but without knowing.

4.3 Primary and secondary endpoints

The main target variables are safety parameters (side effects, infections, lymphoma, and malignancies). Secondary endpoints are efficacy parameters (rejections, patient/graft survival, and lymphocyte depletion).

4.3.1 Primary endpoints

The main target variables are safety parameters. Variables to be assessed are incidences of selected adverse events. The primary outcome measure of the trial is the incidence of the following signs and symptoms (individually and in combination) at any time during randomized treatment and follow-up:

1. Anaphylaxis

2. Serum sickness

3. Two of the following symptoms: Fever >38°, chills, nausea/vomiting or erythema

4. One of the following findings:

- Hematological side effects (leucopenia (<3000), thrombocytopenia (<50’000), hyporegenerative anemia (hemoglobin < 100g/l plus non-elevated reticulocytes or erythropoietin substitution)
- Severe infectious complications defined by prolonged hospital stay, needing ICU or re-hospitalization.
- Lymphoma, post-transplant lymphoproliferative disease (PTLD), malignancy
- Number of patients withdrawn due to adverse events or for other safety-related reasons

→ Primary endpoint is reached when 1 event of 1 to 4 is positive.

Anaphylaxis is a clinical diagnosis consisting of following possible symptoms: elevated termperature, generalized exanthema (flush), edema, dyspnea, stridor and hypotension.

Serum sickness is a clinical diagnosis consisting of following possible symptoms occurring after 6 to 14 days and lasting for 1 to 5 days: sudden fever, generalized exanthema, arthritis, meningitis, nephritis, enteritis and polyserositis.

These symptoms will be evaluated as attributable adverse events if a causal relationship with the investigational treatment cannot be excluded (i. e., causality assessment "probable", "possible" or "unlikely"; see section 7.3 G for details).

4.3.2 Secondary endpoints

- Incidence of acute rejection episodes and type (cellular or humoral) of rejection (according to Banff)
  - Total number and type of anti-rejection treatments
- Graft function
  - Serum creatinine at day 1, 7, 14, and month 1, 3, 6, 12
  - Proteinuria month 1, 3, 6 and 12
- Graft and patient survival

4.4 Time schedule

The recruitment will start in November 2008. The enrolment will continue approximately 24 months until the desired patient number is reached, followed by a 12- month observation period.

Duration of treatment for:
ATG-Fresenius S: 5 days (per protocol)
Thymoglobulin: 4 days (per protocol)

4.5 Discontinuation criteria

a) Individual subjects

Reasons for the discontinuation of the treatment of single patients during or after the study are:

- withdrawal of Informed Consent;
- evident lack of compliance of the patient;
- onset of HIV during the trial period;
- pregnancy;
- not permitted co-medication if it is a safety risk for the patient;
- relevant protocol deviation if it is a safety risk for the patient;
- severe surgical complications during the transplantation if it is a safety risk for the patient;
- lost to follow-up;

Whenever possible, patients in whom the investigational treatment is discontinued prematurely will be followed up until the scheduled end of the period of observation. Unless a patient withdraws his/her informed consent, is lost to follow-up or continued study participation presents an intolerable risk, withdrawal from treatment does not lead to withdrawal from the scheduled examinations or from the trial.

b) Entire trial

The study can be terminated:

- In cases of not previously reported risk, accumulation of serious adverse drug reactions resp. adverse events, relevant protocol deviations. The decision is taken by the investigator.and the respective manufacturer is informed.
- If there are major arguments against continuation, the principal investigator has the right to terminate the study prematurely.

4.6 Drug responsibility

1. ATG-Fresenius S

a) Labelling of the investigational drug:
The label of ATG-Fresenius S will be the commercial label of the product for Switzerland.

b) Specification of the way of delivery and the record of the receipt of medicinal products:
The investigational drug is usually delivered in a commercial manner to the investigator. The investigational drug is produced and stored in Gräfelfing. Following release by Quality Control the drugs are shipped directly to the pharmacy of the investigation center.

c) Regulations concerning the storage in the clinic/practice:
The investigational drug has to be stored protected from light and dry at a temperature of +2° C to +8° C. The investigator is directly responsible for the storage of the investigational drug.

Only collaborators involved directly into this study have access to the investigation drugs. The dispersion and use of the investigational drugs has to be documented on separate form sheets (see enclosure 2).

d) Regulation concerning the return of unused medicinal products at the end of the study or after expiry date, respectively:
As ATG-Fresenius S will be bought in commercial manner, the investigator is responsible for the unused medicinal product. The use of ATG-Fresenius S has to be documented (see enclosure 2).

2. Thymoglobulin

a) Labelling of the investigational drug:
The labelling of Thymoglobulin will be the commercial label of the product for Switzerland.

b) Specification of the way of delivery and the record of the receipt of medicinal products:
The investigational drug is usually delivered in a commercial manner to the investigator. Following release by Quality Control the drugs are shipped directly to the pharmacy of the investigation center.

c) Regulations concerning the storage in the clinic/pratic:
Thymoglobulin is supplied in vials as a sterile, lyophilised powder to be reconstituted with sterile diluent. Vial 1 contains 25 mg freeze-dried rabbit anti-human thymocyte immunoglobulin. Vial 2 contains the diluent. Vials should be stored between the temperatures of +2° to +8°C (36° to 46°F); do not freeze the vials. Protect undiluted solution against direct light. Diluted medication is stable for 24 hours at 4°C or for 4 hours at room temperature.

d) Regulation concerning the return of unused medicinal products at the end of the study or after expiry date, respectively:
As Thymoglobulin will be bought in commercial manner, the investigator is responsible for the unused medicinal product. The use of Thymoglobulin has to be documented (see enclosure 3).

5. SELECTION AND WITHDRAWAL OF SUBJECTS

5.1 Study population

Patient population: Consecutive recipients of a renal allograft who are at high immunological risk and who are at least 18 years or older. Two core centers will initiate the trial but other Swiss and foreign state centers are allowed to take part if they manifest their interest.

5.2 Inclusion criteria

1. Recipients, who are at least 18 years or older and have a high immunological risk defined by:
   The presence of at least one donor-specific antibody (class I and/or II) detected and specified by flow-technology (FlowPRA and single antigen Flow-beads), which are
   - For class I below the threshold of detection of a current CDC T-cell-/ and B-cell cross-match
   - For class II below the threshold of detection of a current CDC B-cell cross-match.
2. Patient receives a renal allograft only.
3. Female patients of child bearing age agree to maintain effective birth control practice during the study.
4. Patient has been fully informed and has given written or independent person witnessed oral informed consent.

5.3 Exclusion criteria

1. Patient is pregnant or breastfeeding.
2. Patient and donor are ABO incompatible.
3. Age of donor >75 years.
4. Patient has leukopenia, defined as having at transplantation less than $3.000/mm^3$ leukocytes.
5. Patient has thrombocytopenia, defined as having at transplantation less than $75.000/mm^3$ thrombocytes.
6. Patient is allergic or intolerant to ATG-Fresenius S, Thymoglobulin, steroids, Tacrolimus, MMF, or IVIG.

11. EBV risk constellation (recipient EBV negative and donor EBV positive).
12. Patient or donor is known to be HIV positive.
13. Patient has significant liver disease, defined as having during the past 28 days continuously ASAT (SGOT) and/or ALAT (SGPT) levels greater than 3fold of the upper value of the normal range of the investigational site.
14. Patient with malignancy or history of malignancy <2 years, except non metastatic basal or squamous cell carcinoma of the skin that has been treated successfully.
15. Patient has significant, uncontrolled concomitant infections and/or severe diarrhea, vomiting, or active peptic ulcer.
16. Patient is unlikely to comply with the visits schedule in the protocol.

6. TREATMENT OF SUBJECTS

6.1 Administration route, single dose, dosage, treatment period, follow-up for subjects

All patients included will provide written informed consent to participate in the trial.

Blood parameters will be determined before study drug administration which will be used as baseline values.

In women of childbearing age a pregnancy test will be performed before inclusion.

Patients qualified for the trial will be randomised to one or the other treatment.

The treatments will be administered as open therapies. A central venous catheter will be placed and kept open using a normal saline or glucose infusion drip or a fistula. This line will be used to administer test treatments.

Intravenous methylprednisolone sodium succinate (see table “therapy scheme I and II”) should be given 1 hour before administering the test drugs.

Patients randomised to receive a bolus therapy with ATG-Fresenius S will be treated i.v. with a 9 mg/kg bw bolus of ATG-Fresenius S, which is diluted in 100-500 ml physiological saline. The infusion has to be applied completely before reperfusion (infusion time at least 1 hour). After this first infusion the patients receive 3 mg/kg bw/d for 4 days given over 4 hours.

Patients randomized to receive Thymoglobulin will be treated i.v. with 1.5 mg/kg bw of Thymoglobulin, which is diluted in 100-500 ml physiological saline. The infusion has to be completed before reperfusion (infusion time at least 1 hour). After this first infusion the patients receive 1.5 mg/kg bw/d for 3 days given over 4 hours.

The patients will be observed closely for the first 2 hours of treatment.

6.2 Transplant monitoring

Standard monitoring:

- The patients will be followed by the routine clinical, biochemical and imaging parameters.
- Blood pressure, heart rate, temperature and urine output will be recorded daily during hospitalisation.
- Standard blood work as routinely done includes:
  - Complete blood count (including hemogram, leukocytes, reticulocytes and platelet count).
  - Complete blood chemistry (including serum creatinine, blood urea nitrogen (BUN), sodium, potassium, calcium, phosphate, uric acid, total protein, albumin, bilirubin, SGOT (AST), SGPT (ALT), LDH, gamma-GT, alkaline phosphatase, glucose, cholesterol and HbA1c.
- Standard blood work will be performed at each visit and at least at days 0, 1, 7, 14 and at months 1, 3, 6, 12 and 24.

Optional monitoring:

- Protocol allograft biopsies will be done on day 7 post-transplant, at month 3, 6, and 12.
- Lymphocyte subpopulations consisting of cells positive for CD3, CD4, CD8, CD19, and CD56 will be measured by flow-cytometry before transplantation, daily for the first 7 days, at month 1, 3, 6, 12, and 24.
- Monitoring for viral infection/reactivation (e.g. Decoy cells in urine, Polyoma-BK-viremia, EBV-viremia, CMV-antigenemia or CMV-viremia).

6.3. Diagnosis and treatment of rejection episodes

Clinical acute rejection episodes are defined by an increase of serum creatinine of >20% from baseline with consisting histology classified according to local practice.

Subclinical acute rejection episodes are defined by a protocol biopsy with consisting histology classified according to local practice.

The treatment of rejections is ad libidum of the physician, however, a cross-over treatment as rescue-therapy by using the immunoglobulin preparation (AFG-Fresenius S or Thymoglobulin) from the other group is prohibited.

6.4. Prophylaxis and treatment of infections

A) Pneumocystis carinii
As prophylaxis it is recommended to administer Cotrimoxazole double strength 1 Tbl. 3x/week for 6 months. Alternatively an inhalation of Pentamidin once a month for 6 months can be given.

B) CMV
CMV constellation R-/D- will not receive prophylaxis. In all other constellations (R+/D-, R+/D+, R-/D+) prophylaxis with valgancyclovir p.o. (900mg/day adapted to renal function) will be done for 3 months.

CMV disease is defined according to Ljungman CID 2002 and Preiksaitis AJT 2004, respectively.

THERAPY SCHEME I
(ATG-Fresenius S GROUP)

Period	ATG-Fresenius S	IVIG	Tacrolimus	MMF	Prednison
**Day -1** (if applicable)	-		0.2 mg/kg/d	1000 mg p.o.	-
**Day 0** (operation)	9 mg/kg bw infusion The infusion has to be completed before reperfusion	0.4 g/kg bw given pre-operatively or post-operatively as soon as possible (maximal total dose 140 g)	0.2 mg/kg/d	2 x 1000 mg/d p.o.	1000 mg Methylprednisolone i.v. Has to be completely infused before ATG-Fresenius S is given
**Day 1**	3 mg/kg bw i.v. *	0.4 g/kg bw	0.2 mg/kg/d	2 x 1000 mg/d p.o	500 mg MP i.v.
**Day 2**	3 mg/kg bw i.v. *	0.4 g/kg bw	0.2 mg/kg/d	2 x 1000 mg/d p.o	250 mg MP i.v.
**Day 3**	3 mg/kg bw i.v. *	0.4 g/kg bw	0.2 mg/kg/d	2 x 1000 mg/d p.o	0.5 mg/kg/d p.o. rounded to 5 mg (downwards)
**Day 4**	3 mg/kg bw i.v. *	0.4 g/kg bw	0.2 mg/kg/d	Dose adjustment to target blood levels and white cell count	Dose adaption (see below)
**Day 5**	-		0.2 mg/kg/d then adjust to target blood levels (see below)	Dose adjustment to target blood levels and white cell count	Dose adaption (see below)
**Day 6**	-		Adjusted to target blood levels (see below)	Dose adjustment to target blood levels and white cell count	Dose adaption (see below)

* (dose adaptation see below)

THERAPY SCHEME II
(Thymoglobulin GROUP)

Period	Thymoglobulin	IVIG	Tacrolimus	MMF	Prednison
**Day -1** (if applicable)	-		0.2 mg/kg/d	1000 mg p.o.	-
**Day 0** (operation)	1.5 mg/kg bw infusion The infusion has to be completed before reperfusion	0.4 g/kg bw given preoperatively or postoperatively as soon as possible (maximal total dose 140g)	0.2 mg/kg/d	2 x 1000 mg/d p.o.	1000 mg Methylprednisolone i.v. Has to be completely infused before Thymoglobulin is given
**Day 1**	1.5 mg/kg infusion (dose adaptation see below)	0.4 g/kg bw	0.2 mg/kg/d	2x 1000mg/d p.o.	500 mg MP i.v.
**Day 2**	1.5 mg/kg infusion (dose adaptation see below)	0.4 g/kg bw	0.2 mg/kg/d	2x 1000mg/d p.o.	250 mg MP i.v.
**Day 3**	1.5 mg/kg infusion (dose adaptation see below)	0.4 g/kg bw	0.2 mg/kg/d	2x 1000mg/d p.o.	0.5 mg/kg/d p.o. rounded to 5 mg (downwards)
**Day 4**	-	0.4 g/kg bw	0.2 mg/kg/d	Dose adjustment to target blood levels and white cell count	Dose adaption (see below)
**Day 5**	-		0.2 mg/kg/d then adjust to target blood levels (see below)	Dose adjustment to target blood levels and white cell count	Dose adaption (see below)
**Day 6**	-		Adjusted to target blood levels (see below)	Dose adjustment to target blood levels and white cell count	Dose adaption (see below)

C) Dose adaptations

a) ATG-Fresenius S and Thymoglobulin dose adaptations

Reduce to half the dose when leucopenia (2.000-3.000/mm^3) and/or thrombocytopenia (50.000-75.000/mm^3) are present

Withhold either drug when leukopenia (<2.000) and/or thrombocytopenia (<50.000) are present

b) Tacrolimus target trough blood levels

**Period**	**Whole blood 12 h trough level**
Day 3 or 4 onwards, kidney well functioning	10 -12 ng/ml
Month 3 onwards, kidney well functioning, normal protocol biopsy	8 - 10 ng/ml
Month 6 onwards, kidney well functioning, normal protocol biopsy	6 - 8 ng/ml
Month 12 onwards, kidney well functioning, normal protocol biopsy	4 - 6 ng/ml
Rejection	Increase by 2 - 5 ng/ml
Drug-toxicity (e.g. nephrotoxicity, infections, increased viral replication (i.e. EBV, Polyoma-BK))	Decrease by 2 - 5 ng/ml

c) Dose adjustment of MMF according to blood levels and leukocyte count

**Period**	Dose adjustment of MMF according to blood levels and leukocyte count
From day 4 onwards	Blood levels should be preferably between 2 - 4 ng/ml
From day 4 onwards	If leukocyte counts are below 3.500 mm^3: MMF has to be reduced according to the judgement of the treating physician
From day 4 onwards	If neutrophil granulocytes are below 800/mm^3: MMF has to be stopped

d) Tapering steroid dosage

Starting at day 3 with 0.5 mg/kg bw, tapered by 5 mg biweekly until 15 mg, then by 2.5 mg biweekly until 0.1 mg/kg until month 6. In case of rejection, steroids may be increased at the discretion of the responsible physician. Steroids can be withdrawn after month 6 at the discretion of the responsible physician.

6.5 Procedures for monitoring subject compliance

ATG-Fresenius S and Thymoglobulin must only be administered according to the instructions of a physician and under medical supervision. This avoids a deviation from compliance by the patient. The physician in charge is responsible to document the applied amount of ATG-Fresenius S/ Thymoglobulin in the CRFs.

6.6 Handling of the investigational drug

a) Rules for the use of concomitant treatment:
Sodium Heparin must not be added to the ATG-Fresenius S infusion solution or administered via the same route, because a pH below 5.2 may lead to turbidity.

b) Measures to be implemented to ensure the safe handling of the drugs, measures to control close adherence to the instructions:
ATG-Fresenius S is a hypotonic concentrate for infusion with pH 3.7. Before intravenous infusion it must be diluted with physiological saline solution.
ATG-Fresenius S must only be administered according to the instructions of a physician and under medical supervision. Particular attention must be paid to monitoring the patient for any symptoms of anaphylactic shock. Close monitoring of the patient must continue for at least 30 minutes following the end of the infusion.

For peripheral administration a large vessel should be chosen. It has been found that systemic tolerance is improved, if hydrocorticosteroids and/or antihistaminic drugs are administered prior to infusion.

c) Thymoglobulin
The powder should be mixed with the solvent that is added in the package. Thymoglobulin is infused after dilution in saline (0.9%) or dextrose (5%) solution (per one vial use 50 ml of infusion solution). Mix the solution by inverting the bag gently once or twice.

Infuse slowly into a high-flow vein. Adjust the infusion rate so that the total duration of infusion is at least one hour for the first dose and over at least 4 hours for subsequent doses.

Instructions for Administration:

The recommended route of administration is intravenous infusion using a high-flow vein and should be infused over at least one hour for the first infusion and over at least 4 hours on subsequent days. The calculated number of Thymoglobulin vials should be transferred into a bag of infusion solution (0.9% saline or 5% dextrose) before intravenous administration to patients. When mixing the solution, gently invert the bag only one or twice: DO NOT SHAKE.
Inspect solution for particulate matter after reconstitution. Should some particulate matter remain, continue to gently rotate the vial until no particulate matter is visible. It particulate matter persits, discard this vial.

Do not use Thymoglobulin after expiration date indicated on the label.

Any unused portion of the drug must be discarded.

Once the infusion is prepared, it should be used within 4 hours after reconstituting if kept at room temperature.

7. ASSESSMENT OF SAFETY

7.1 Specification of safety parameters

All adverse events reported will be included in the safety evaluations with particular attention to the list mentioned in chapter 4.3.1. All symptoms will be inquired actively.

7.2 Methods and timing for assessing, recording, and analysing of safety parameters

Adverse events, reported by the patient and/or observed by study personnel, will be summarized for each treatment group. Laboratory data will be compared to the corresponding normal ranges. Physiopathologically relevant high or low parameters will be summarized for each treatment group. Per protocol all AE documentation is performed continuously.

7.3 Procedures for eliciting reports of and for recording and reporting adverse events and intercurrent illnesses

Definitions:

a) Adverse drug reaction: A response to a drug which is noxious and unintended and which occurs at doses normally used in man for prophylaxis, diagnosis or therapy of disease or for modification of physiological function.
It should be considered, that „death" per se is no adverse drug reaction, but may be the consequence or the outcome of an event, which may be an adverse drug reaction and which should be assessed. If the reason of death is unknown and remains unknown despite proper investigation, the phrase „inexplicable death" may be used to describe the reaction.

b) Serious adverse drug reaction
A serious adverse drug reaction is a drug reaction, which results in death, is life-threatening, requires inpatient hospitalisation or prolongation of existing hospitalisation, or results in persistent or significant disability/incapacity.

c) Unexpected adverse drug reaction
An unexpected adverse drug reaction is an adverse drug reaction, which is not mentioned in the Summary of Product Characteristics, the package insert, the Scientific Brochure, the Investigator's Brochure, the protocol or elsewhere.

d) Unexpected serious adverse drug reaction
An unexpected serious adverse drug reaction is defined as an adverse drug reaction, which is unexpected as well as serious.

e) Adverse event
An adverse event is any untoward medical occurrence in a patient, who was treated with a pharmaceutical product, independent if there is a causal relationship with the administration of the drug suspected or not.

f) Serious adverse event
Serious adverse events are adverse events, which result in death, are life-threatening, require inpatient hospitalisation or prolongation of existing hospitalisation, result in persistent or significant disability/incapacity. A congenital anomaly/birth defect or malignancy are usually be considered serious.

g) Causality assessment
The causal relationship to the study medication has to be assessed for every adverse event using one of the following terms:

1 = probable
- reasonable temporal sequence after administration of study medication
- the AE has already been described or can be expected as a side effect of the drug
- decrease or disappearance of symptoms after discontinuation of treatment or dose reduction
- renewed appearance of the AE after repeated exposure to the study medication
- no reasonable explanation of the AE via the patient's clinical condition

2 = possible
- reasonable temporal sequence after administration of study medication
- the AE has already been described or can be expected as a side effect of the drug
- the AE may be caused by several other reasons

3 = unlikely
- reasonable temporal sequence after administration of study medication
- the AE has not yet been reported as a side effect of the drug and cannot be expected
- the AE remains persistent after discontinuation of treatment or dose reduction
- repeated exposure to the study medication does not lead to the same event
- the AE may be caused by several other reasons

4 = no relationship
- no reasonable temporal sequence after administration of study medication
- the AE has obviously been caused by other reasons, e.g. symptoms of the study indicating disease itself

5 = assessment impossible
- the available data are insufficient for any reasonable assessment with respect to a causal connection

In this open-label trial the causality assessments of all adverse events will be provided by blind raters. The rater will receive all information available for a particular adverse event, including predisposing factors from the patients’ history, concomitant medication and diseases, but he/she will be unaware of the investigational treatment actually received. For each adverse event two different causality assessments will be obtained: (a) assuming that the patient has received ATG and (b) assuming that he/she has received Thymoglobulin. During data analysis only the assessment corresponding to the treatment actually received will be used.

All cases where 1, 2 or 3 applies will be evaluated as attributable adverse events / ADRs.

All adverse events, i. e. also the already known ADRs, must be documented and serious adverse events also be registered. There is also an obligation to registration if a causal relationship is not supposed resp. not evident. The following items must be registered:

- Precise trial subject data;
- precise description of the AE;
- precise recording of the time of the AE in relation to the course of therapy;
- intensity of the AE;
- results of diagnostic and therapeutic measures,
- where applicable, rechallenge and/or dechallenge data;
- course and outcome of the AE;
- severity of the AE, including medical assessment;
- all other important data that may be of relevance for assessment of a possible relationship between the AE and the administration of the investigational medical substance.

Data are documented and registered on the form "adverse events" or „serious adverse events“ (see enclosure 4), whereby the needed additional information can be supplied without a specific form.

h) Intensity assessment

The intensity of an AE is to be graded using the terms mild, moderate, and severe. These are defined as follows:

Mild: the event is realized but well tolerated
Moderate: the event interferes with usual daily activities
Severe: the event makes usual daily activities impossible

Note: A strict difference is to be made between intensity grading and whether an adverse event is serious or not. A highly pronounced AE needs not necessarily be serious.

i) Suspected unexpected serious adverse reaction
As a minimum requirement Serious Adverse Events which are unexpected and related (SUSARs) and Adverse Events which may constitute hazard or safety of subjects recruited in the project shall immediately be reported by the Investigator to the EC, the local Health authority and to the respective local company if applicable.

According to valid regulatory requirements, the respective company has to collect, assess and report Adverse Events, noted in connection with the use of the company products. Therefore, the Investigator shall notify the respective company about all Adverse Events after completion of the project.

7.4 Type and duration of the follow-up for subjects after adverse events

Participants of the trial will be treated according to the physician's judgement. The measures and the outcome of an adverse event (until the end of the follow-up stated in the protocol) have to be documented using the corresponding forms.

7.5 Report of serious adverse events

The investigator is responsible to report all serious adverse events (SAEs) within 24 hours by telephone or fax using a SAE report form to the respective drug company.

The initial SAE report should contain the following details: study number, patient number, initials, sex, date of birth, drug allocation, date of onset, description of the event, name, address and telephone number of the investigator.

The investigator is responsible to inform the Ethics Committee of SAEs on an ongoing basis.

The single case documentation of a serious adverse drug reaction, an interaction or an observed, relevant abuse is a report, which has to be submitted within 24 hours by telephone or fax using a SAE report form to the respective drug company. This report is required, if - in relationship with the administration of a drug - an event occurs, which causes a serious reaction (in the sense of the above definition) in a patient.

Following information is considered to be minimal criteria for a single case documentation:
- identified patient;
- suspected drug;
- observed serious adverse drug reaction or interaction/abuse;
- identified data source.

Adverse events, which are not serious, are summarized at the end of the trial in the medical report, but will not be documented on the single case form or reported to the responsible authorities.
Acute steroid sensitive rejections are not considered as serious adverse events despite necessity of hospitalisation, because the events are expected during such a trial. Nevertheless, acute steroid resistant rejections will be notified to the health authorities.

8. ASSESSMENT OF EFFICACY

See 4.3.2 Secondary endpoints

9. STATISTICS

In this pilot study all statistical analyses will be descriptive and p-values will be interpreted accordingly. Confidence intervals will be provided to assist the interpretation of the observed effects.

The primary analysis of safety will assess the incidence of adverse drug reactions (ADRs) attributable to the investigational treatments according to the causality assessment detailed in Section 7.3, paragraph G. A separate analysis will be performed for each of the ADRs listed in Section 4.3.1 as well as for a summary of all ADRs not included in this compilation.

For comparison between ATG and Thymoglobulin the risk ratio (ratio of proportions of patients reporting a particular ADR at least once under trial drug and reference treatment) and the risk difference (difference between proportions) will be determined, including their 95% confidence intervals determined according to the method described by Newcomb (25). For comparisons between the AE rates under both treatments, descriptive two-sided p-values will be determined with Fisher's exact test. In a working definition, the following findings will be considered to be indicators of a potentially meaningful treatment group difference regarding ADR incidence[1]:

- two-sided p-value ≤ 0.15 for the comparison of AE rates,
- risk ratio ≥ 0.5 or ≤ 2.0,
- 95% confidence interval for a risk ratio which excludes the value 1.0, and
- 95% confidence interval for a risk difference which excludes the value 0.0.

All analyses of safety will be performed primarily in the safety data set which includes all patients who have received the investigational treatment at least once. To assess potential bias introduced by premature withdrawal from the trial a sensitivity analysis will be performed in a subset which includes all patients who complete the follow-up period as scheduled (but independently of the presence or absence of protocol violations).

For the analysis of treatment efficacy actuarial life tables will be computed for the following parameters: graft survival, patient survival, time to first rejection episode. All life tables will be based on a period of observation of 24 months.

[1] Relevance criteria derived from (26).

In addition, inferential statistics will be computed for the following parameters:

- Time to 1st and 2nd rejection (Mann-Whitney U-Test).
- Incidence of rejection episodes (Chi-square statistic).
- Number of rejections per patient (Mann-Whitney U-Test).
- Duration of rejection episodes (t-test).
- 12, and 24-months graft survival rate (Chi-square statistic).
- 12 and 24-months patient survival rate (Chi-square statistic).
- Number of prednisone pulses within 12 and 24 months (Mann-Whitney U-Test).
- Rejection reversal rate (Chi-square statistic).

The analysis of efficacy will be based on the full analysis set (FAS) which includes all patients who were randomized and treated and for whom any post-treatment efficacy data are available.

After completion of the 24-months follow-up period the final biometrical analysis will be performed.

Furthermore, all patient data recorded in the trial database will be tabulated by treatment group using methods of descriptive data analysis, with specification of summary statistics applicable to their levels of measurement.

Sample size estimation:
In this pilot study the determination of the sample sized is based primarily on feasibility rather than on statistical reasons. A total of 40 (Zürich 20, Basel 20) randomised and treated patients (20 in each treatment group) will be included.

10. ETHICS AND LEGAL REQUIREMENTS

10.1 Description of ethical considerations relating to the trial

The study protocol will be submitted to the local ethical committees of each participating center. The written approval/positive opinion of the committee is a prerequisite for the start of the study. The current version of the ethical convention is enclosed (see enclosure 5).

10.2 Patient Informed Consent

The prerequisite for the inclusion of a patient into the study is the consent after being informed about the nature, the significance and the consequences of the study, about adverse drug reactions and risks as well as about the possibility of withdrawal from the study at any time. The investigator has to inform the patient. Consent must be given by signature on the respective form (see enclosure 6), confirming the receipt of the information form (see enclosure 6). Furthermore, the patient has to agree in writing, that the data will be recorded, checked for accuracy by a representative of the sponsor and, if applicable, inspected by the responsible authorities (see enclosure 6). The patient has to date the Consent Form personally. The investigator has to hand over a copy of these documents to the patient.

If there are justifiable reasons not to obtain the Informed Consent (only in accordance with current law), the reasons have to be documented.

10.3 Notification

This trial will be notified to Swissmedic or other authorities of foreign state centers, if applicable, by the sponsor. Trial relevant documents (protocol including enclosures and nomination of the principal investigator and trial center, Ethics Committee approval; if applicable the documentation of the drugs' chemical, pharmaceutical, pharmacological, and toxicological properties) will be notified to the responsible authorities.

11. DATA HANDLING AND RECORD KEEPING

11.1 Direct access to source data/source documents

With signature on the page „Responsibilities" the investigator/institution permits a trial related monitoring, auditing, ethics committee review, and regulatory inspection(s) and provides direct access to the source data/source documents.

11.2 Identification of source data

Medical source records are expected for all patient data recorded in the study database.

11.3 Registration of data

For each patient enrolled into the trial a separate CRF has to be used. For this study paper CRFs will be used (see enclosure 7).

All corrections on a CRF and elsewhere in the hard copy raw data must be made in a way which does not obscure the original entry. The correct data must be inserted with the reason for correction, dated and initialled by the investigator.

The CRF must be filled out completely; missing data have to be substantiated. (Each completely filled out CRF has to be signed and dated by the investigator). The investigator states by signature, that all data are correct, complete, and properly ascertained.

Laboratory values with normal reference ranges should always be recorded on CRF or attached to it. Values outside a clinically accepted reference range or values that differ importantly from previous values must be evaluated and commented upon by the investigator.

Data other than those requested by the protocol may appear on the CRF clearly marked as additional findings, and their significance should be described by the investigator.

Units of measurement must always be stated, and transformation of units must always be indicated and documented.

The investigator should always make a confidential record to allow the unambiguous identification of each patient.

11.4 Record of the termination of the study

If the study is terminated in cases of single patients (drop-outs) the reason must be documented and assessed.

11.5 Record keeping

Essential documents should be retained until at least 2 years after the last approval of a marketing application in an ICH region and until there are no pending or contemplated marketing applications in an ICH region or at least 2 years have elapsed since the formal discontinuation of clinical development of the investigational product. These documents should be retained for a longer period, however, if required by the applicable regulatory requirements or by an agreement with the sponsor. It is the responsibility of the sponsor to inform the investigator/institution as to when these documents no longer need to be retained.

11.6 Monitoring

During the study a monitor (a study independent physician) will keep personally contact to the investigator and other persons involved in the study and assure the following commitments:
- obedience to the protocol, especially inclusion and exclusion criteria;
- obtaining the signed informed consent;
- correct keeping of CRFs;
- data check;
- documentation of delivery and use of medical products;
- correct storage of medical products;
- correct report of adverse events.

The monitoring intervals are dependent on the rate of recruitment.

11.7 Publication

Each investigator (Basel and Zürich) can publish the data of their own patients. To publish the data from all patients all investigators involved have to agree.

12. INSURANCE

The subjects of this study are in the case of damage insured by the „Zürich" Versicherungsgesellschaft, 8085 Zürich. . The valid contractual conditions apply.

13. LITERATURE

(1) Gaber AO, First MR, Tesi RJ, et al., (1998) Results of the double-blind, randomized, multicenter, phase III clinical trial of Thymoglobulin versus Atgam in the treatment of acute graft rejection episodes after renal transplantation. Transplantation (1998);66(1):29-37.

(2) Bock, HA, Gallati, H, Zurcher, RM, Bachofen, M, Mihatsch, MJ, Landmann, J, Thiel, G.
Randomized prospective trial of prophylactic immunosuppression with ATG-Fresenius versus OKT3 after renal transplantation.
Transplantation (1995); 59: 830-840.

(3) Djamali, A, Turc-Baron, C, Portales, P, Leverson, G, Chong, G, Clot, J, Mourad, G.
Low dose antithymocyte globulins in renal transplantation.
Transplantation (2000); 69: 799-805.

(4) Ducloux, D, Kazory, A, Challier, B, Coutet, J, Bresson-Vautin, C, Motte, G, Thalamy, B, Rebibou, J-M, Cilalopin, J-M.
Long-term toxicity of Antitymocyte globulin induction may vary with choice of agent: A single-center retrospective study.
Transplantation (2004); 7:1029-1033.

(5) Flechner, SM.
Current status of renal transplantation.
Urol. Clin. North Amer. (1994); 21: 265-283.

(6) Frey, D, Matas, A, Canafax, E, et al.
Sequential therapy: A prospective, randomized trial of MALG vs. OKT3 for prophylactic immunosuppression in cadaveric renal allograft recipients.
Transplantation (1991); 54: 50-56.

(7) Gaston, RS, Shroyer, TW, Hudons, SL, et al.
Renal retransplantation: the role of race, quadruple immunosuppression and flow cytometry cross-match.
Transplantation (1994); 57: 47-54.

(8) Kaden J, May G, Schonemann C, et al.
Effect of ATG prophylaxis in sensitized and non-sensitized kidney graft recipients.
Transplant International (1992); 3: 75-78.

(9) Kaden J, May G, Muller P, et al.,
Intraoperative high-dose anti-T-lymphocyte globulin bolus in addition to triple-drug therapy improves kidney graft survival.
Transplantation Proceedings (1995); 27:1060-1061.

(10) Kaden J, May G, Strobelt B, Müller P.

Intraoperative t-cell depletion prior to completion of anastomoses by high dose single ATG Bolus as a new approach to improve long term results after kidney transplanation.
Transplant Proc (1997); 29: 343-347.

(11) Norrby, J, Olausson, M.
A randomised clinical trial using ATG-Fresenius or ATG Merieux as induction therapy in kidney transplantation.
Transplant Proc (1997); 29: 3135-3136

(12) Pascual, J, Marcan, R, Orofino, L, et al.
Evidence that addition of azathioprine improves renal function in CsA treated patients.
Transplantation (1991); 52: 276-279.

(13) Richardson, AG, Higgins, RM, Ratcliff, PF, et al.
Triple therapy immunosuppression in cadaveric renal transplantation.
Transpl. Int. (1990); 3: 26-31.

(14) Saint-Hillier, Y, Hory, B, Racadot, E, et al.
Systemic IL-10 release after a single pre- or peroperative large dose of ATG-Fresenius in human kidney transplantation.
Fresenius Literature Citation (1994); CITIC.

(15) Saint-Hillier, Y, Fournier, V, Racadot, E, Hory, B, Bresson-Vautrin, C.
Pretransplant booster dose of a polyclonal T-activated antibody: A step towards induction of tolerance in human kidney transplantation.
CITIC (1997); Lyon

(16) Saint-Hillier, Y, Racadot, E, Fournier, V, Hory, B, Bresson-Vautrin, C, Jamali, M Ducloux, D, Rebibou, JM, Vautrin, P, Bittard, H, Laplaza, D, Morel, MH, Chabod, J, Tiberghien, P, Chalopin, JM.
Pretransplant bolus induction: Impact of two different polyclonal antibodies, ATG-Fresenius versus Thymoglobulin, during five years in kidney transplantation.
Jurata (2000);

(17) Shackleton, CR, Keown, P, McLaughlin, MB, et al.
The quality of initial function following renal transplantation determined by creatinine elimination kinetics: comparison of MALG and cyclosporin induction immunosuppression.
Transplantation (1991); 52: 1008-1012.

(18) Sinha, SK, Todd, SC, Hedrick, JA, Speiser, CL, Lambris, JD, Tsoukas, CD.
Characterization of the EBV/C3d receptor on the human Jurkat T cell line: evidence for a novel transcript.
The journal of Immunology (1993); 12: 5311-5320.

(19) Sommer, B, Henry, M, Ferguson, R.

Sequential antilymphoblast globulin and cyclosporin for renal transplantation.
Transplantation (1987); 43: 85-90.

(21) Strata, RD, Allesandro, A, Armbrust, M, et al.
Sequential antilymphoblast globulin/cyclosporin in cadaveric renal transplantation: effect of duration of ALG therapy.
Transplantion (1989); 47: 96-102.

(22) Thervet, E, Chatenoud, L, Legendre, C, Mamzer-Bruneel, M-F, Kreis, H, Bach, JF.
Clinical significance of T-Cell monitoring during Antitymocyte globulin induction in renal transplantation.
Transplant Proc (1997); 29: 2330-2331.

(23) van Dorp, WP, van ESL, Thompson, J.
The effect of maintenance immunosuppressive protocol after renal transplantation on infectious complications.
Transplantation (1991); 51: 193-197.

(24) Zietze, R, von Steenberge, EP, Hesse CJ, et al.
Single shot high dose rabbit ATG for rejection prophylaxis after kidney transplantation.
Transpl. Int. (1993); 6: 337-340.

(25) Newcombe RG (1998) Interval estimation for the difference between independent proportions: comparison of eleven methods. Stat Med 17: 873-890

(26) Trautmann-Sponsel RD, Dienel A (2004) Safety of Hypericum extract in mildly to moderately depressed outpatients: a review based on data from three randomized, placebo-controlled trials. J Affect Disord 82: 303-307

14. ENCLOSURES

The protocol comprises the following supplements:

1. Declaration of Helsinki
2. Form for documentation of receipt, dispersion, and return of the investigational substance;
3. Documentation of unused investigational substance;
4. Information form “Measures in cases of Adverse Events”, form “AE”, “SAE”;
5. Ethic convention;
6. Text of the written information and consent form for the trial subjects;
7. Case report form (CRF);
8. Copy of certificate of insurance for the trial subjects;

The enclosures are numbered according to their appearance in the protocol. A reference in the corresponding passages of protocol points to the relevant enclosure.
