# Supplementary material for: A Comparison of Two Types of Rabbit Antithymocyte Globulin Induction Therapy in Immunological High-Risk Kidney Recipients: A Prospective Randomized Control Study
Source: PLoS One. 2016 Nov 17;11(11):e0165233. doi: 10.1371/journal.pone.0165233 (PMC5113896; doi:10.1371/journal.pone.0165233)
Supplement: S1 Table — (PDF) [file pone.0165233.s003.pdf]

Random Number	group	age	gender	kidney disease	number of transplantation	CMV status	Donor age	gender	donor type	ECD	ischemia time min.
100001	T	28.28	m	prim. FSGS	3	pos	57	m	deceased	n	508
300001	A	35.70	m	HUS	2	neg	15	f	deceased	no	300
100003	T	42.72	f	GN	1	neg	14	m	deceased	no	450
300002	T	67.45	f	ADPKD	1	pos	63	m	deceased	yes	1020
300003	T	42.78	m	GN	2	pos	64	f	deceased	yes	480
300004	A	53.74	m	ADPKD	1	pos	50	f	living	n/a	105
300005	A	49.77	m	ADPKD	1	pos	65	m	deceased	yes	803
100004	A	44.59	f	ANCA	2	neg	63	m	deceased	yes	300
300006	T	52.29	f	Reflux	2	pos	69	f	deceased	yes	111
100005	A	67.59	m	ADPKD	1	neg	37	m	deceased	no	1200
300007	A	47.49	f	SLE	2	pos	17	m	deceased	no	570
100007	T	34.81	m	CAKUT	4	neg	65	f	living	n/a	113
100008	A	57.01	m	IgA	2	pos	66	m	deceased	yes	1152
100009	T	51.87	m	IgA	1	pos	67	f	living	n/a	125
100010	A	68.73	f	ADPKD	1	neg	69	m	living	n/a	90
100011	A	62.95	f	ADPKD	1	pos	46	m	living	n/a	90
100012	T	58.43	m	GN	2	neg	55	f	living	n/a	98
100013	A	45.81	m	vasc.GN	1	pos	54	m	deceased	no	646
100014	T	46.47	m	GN	2	neg	59	f	deceased	no	720
300009	T	49.09	f	ADPKD	1	pos	42	f	living	n/a	90
300010	A	61.68	f	IgA	1	pos	8	f	deceased	no	1038
300012	T	54.37	m	diabet.GN	3	pos	48	f	deceased	no	450
300011	A	39.78	m	ADPKD	1	pos	63	f	living	n/a	140
100015	T	44.48	f	GN	3	pos	37	m	deceased	no	420
100016	A	56.38	m	Fabry	2	neg	70	m	deceased	yes	898
100017	A	59.43	m	GN	2	neg	56	f	deceased	no	341
100018	A	65.63	m	ADPKD	2	neg	69	f	deceased	yes	653
100019	T	56.72	m	IgA	2	neg	55	f	living	n/a	49
100020	T	69.65	f	ADPKD	2	pos	50	f	deceased	yes	1220
100021	A	53.98	f	IgA	1	pos	11	f	deceased	no	
100022	T	69.63	m	IgA	2	pos	57	f	deceased	no	665
100023	A	51.23	m	diabet.GN	1	neg	50	f	deceased	no	76
100024	T	56.11	f	GN	3	neg	69	m	deceased	yes	749
100025	A	62.33	f	diabet.GN	1	pos	68	f	deceased	yes	436
300013	T	41.74	m	vasc. GN	1	neg	15	m	deceased	no	583

Random Number	Mismatches	number of DSA	Class I only	Class II only	Class I+II	Class I	Class II	cumulativ Highest MFI	cumulativ pre-Tx MFI	single highest MFI	Highest MFI	pre-Tx MFI	CMV Risk D+/R-	CMV D+/R+; D-/R+	CMV D-/R-	CMV donor	EBV donor
100001	5	1	0	1	0		DR53	7266	7266	7266	7266	7266	0	1	0	neg	pos
300001	4	1	1	0	0	A1		895	0	859	859	0	0	0	1	neg	pos
100003	2	2	0	0	1	A24	DQ6	8600	8600	7400	1800, 7400	1200, 7400	0	0	1	neg	pos
300002	4	1	1	0	0	A29		3014	920	3014	3014	920	0	1	0	pos	pos
300003	3	3	0	1	0		DR13, DQ 6,7	33112	21308	12489	1839 ,12489, 8784	11839, 685, 8784	0	1	0	pos	pos
300004	5	1	1	0	0	A25		4110	4110	4110	4110	4110	0	1	0	pos	pos
300005	4	3	0	1	0		DR4, DQ5, DQ8	4815	0	2288	1126, 2288, 1401	0,0,0	0	1	0	pos	pos
100004	4	1	1	0	0	A68		2004	1617	2004	2004	1617	1	0	0	pos	pos
300006	2	1	1	0	0	B50		886	0	886	886	0	0	1	0	neg	pos
100005	3	1	1	0	0	CW6		3998	3998	3998	3998	3998	0	0	1	neg	pos
300007	2	2	0	0	1	B51	DR7	8780	397	7530	7530, 1250	234, 162	0	1	0	pos	pos
100007	1	1	1	0	0	B62		5000	0	5000	5000	0	0	0	1	neg	pos
100008	5	2	0	0	1	Cw6	DQ2	7518	7518	6977	6977, 592	6977, 541	0	1	0	neg	pos
100009	2	2	0	0	1	A26	DQ6	2500	2500	1300	1300, 1200	659, 964	0	1	0	pos	pos
100010	5	1	1	0	0	CW 17		8910	8910	8910	8910	8910	0	0	1	neg	pos
100011	4	3	0	0	1	B60	DR1, DQ5	10555	6573	7355	7355,1643,1557	4434, 1635, 504	0	1	0	pos	neg
100012	3	1	0	1	0		DR 11	1100	0	1100	1100	0	1	0	0	pos	pos
100013	5	1	0	1	0		DQ9	1115	1115	1115	1115	1115	0	1	0	pos	pos
100014	3	1	1	0	0	B57		2625	1623	2625	2625	2625	1	0	0	pos	pos
300009	5	1	1	0	0	B62		994	994	994	994	994	0	1	0	pos	pos
300010	3	4	0	0	1	A3, B38	DR7, DQ2	12436	8285	6389	3084 , 1622, 1341	6389, 1672 , 113, 111	0	1	0	pos	pos
300012	2	1	0	1	0		DQ 6	6298	6298	6298	6298	6298	0	1	0	pos	pos
300011	3	1	1	0	0	B51		1243	249	1243	1243	249	0	1	0	pos	pos
100015	3	1	1	0	0	CW 4		1965	1711	1965	1965	1711	0	1	0	neg	pos
100016	2	2	1	0	0	A31, CW1		2665	911	1567	1098, 1567	0, 911	0	0	1	neg	pos
100017	4	2	0	1	0		DQ4, DQ8	28549	28549	15530	28549	28549	1	0	0	pos	pos
100018	1	1	1	0	0	B62		2416	2416	2416	2416	2416	1	0	0	pos	pos
100019	4	1	0	1	0		DP9	7900	0	7900	7900	7900	1	0	0	pos	pos
100020	4	2	1	0	0	B38, B44		14917	14917	7499	7499, 7418	7499, 7418	0	1	0	pos	pos
100021	6	1	1	0	0	CW4		774	666	774	774	666	0	1	0	pos	pos
100022	3	1	0	1	0		DQ9	4074	1754	4074	4074	1754	0	1	0	pos	pos
100023	4	1	0	1	0		DRB1	983	684	983	983	684	1	0	0	pos	pos
100024	1	1	1	0	0	A32		5163	5163	5163	5163	5163	1	0	0	pos	pos
100025	4	2	0	1	0		DR 17, DQ7	2399	2399	1405	994, 1405	994, 1405	0	1	0	neg	pos
300013	3	1	0	1	0		DQ2	880	570	880	880	570	0	0	1	neg	neg

Random Number	viral infections EBV (first 6mo)	CMV (first 6mo)	Herpesvirus (HSV,VZV)	bacterial infections Overall	UTI	Pyelonephritis	wound infection	others	funguus	Hb day 0	WBC day 0	Neutro day 0	Lymp day 0	Tc day 0
100001	0	0	0	0	0	0	0	0	0	107	4600	2654	1140	302
300001	1	0	0	0	0	0	0	0	0	125	8100	5900	90	146
100003	0	0	0	1	1	0	0	0	0	111	8100	5863	893	319
300002	0	1	0	1	0	0	1	0	0	114	6000	4300	1200	147
300003	0	1	0	1	0	0	0	1	0	124	6100	4300	1200	265
300004	1	0	0	0	0	0	0	0	0	112	5000	3200	1100	253
300005	0	0	0	0	0	0	0	0	0	123	3200	1500	1200	193
100004	0	0	0	1	0	1	0	0	0	134	9200	7067	551	295
300006	0	1	0	3	2	0	0	1	0	112	4500	2600	1300	181
100005	0	0	0	1	0	0	0	1	0	105	4200	3050	308	149
300007	0	0	0	2	2	0	0	0	0	110	6400	3800	1500	266
100007	0	0	0	0	0	0	0	0	0	94	6850	6103	390	172
100008	0	0	1	0	0	0	0	0	0	131	7170	5069	1563	137
100009	0	0	0	0	0	0	0	0	0	129	8000	4888	2272	212
100010	0	0	0	6	4	1	0	1	1	114	5870	4919	816	150
100011	0	1	0	1	1	0	0	0	1	108	7600	5784	1440	177
100012	0	0	0	0	0	0	0	0	0	110	8920	6306	1427	456
100013	0	0	0	0	0	0	0	0	0	110	11300	9413	1013	308
100014	0	1	0	0	0	0	0	0	0	98	11650	7969	1060	437
300009	0	0	0	0	0	0	0	0	1	120	5500	3200	1800	156
300010	0	1	0	0	0	0	0	0	1	143	6700	4200	1800	217
300012	0	0	1	1	1	0	0	0	0	122	3600	1900	1100	180
300011	0	0	0	2	0	0	0	2	0	124	5200	2800	1900	145
100015	0	0	0	0	0	0	0	0	0	104	6870	4570	1520	266
100016	0	0	1	1	0	0	0	1	0	109	5940	5520	750	210
100017	1	0	0	0	0	0	0	0	0	130	8420	5313	2189	224
100018	0	0	0	4	3	0	1	0	0	135	3700	2700	590	126
100019	0	0	0	0	0	0	0	0	0	90	7870	7240	189	374
100020	0	0	0	1	0	0	1	0	0	109	4700	2950	1060	138
100021	0	0	0	0	0	0	0	0	0	123	4580	3188	861	236
100022	0	0	0	0	0	0	0	0	0	100	5040	3936	600	111
100023	1	0	0	1	1	0	0	0	0	125	7380	5350	1277	260
100024	0	0	0	7	7	0	0	0	0	122	5840	3866	981	262
100025	1	0	0	1	1	0	0	0	0	114	9200	6150	1540	373
300013	0	0	0	0	0	0	0	0	0	131	5200	3800	900	209

Random Number	Hb day 1	Hb delta	WBC day 1	Neutro day 1	Lymp day 1	Tc day 1	Krea day 1	GFR day 1	Ec- Substitution day 0-14	DGF	Hb day 7	WBC day 7	Neutro day 7	Lymph day 7	Tc day 7
100001	80	27	8700	8390	87	171	676	9.02	yes	yes	86	3900	3073	341	152
300001	91	34	4600	4200	200	125	943	5.86	no	yes	75	3700	2300	600	138
100003	108	3	17700	17255	124	218	867	6.23	yes	no	92	6200	5314	405	184
300002	66	48	15900	15700	300	114	381	14.66	yes	no	72	10800	9300	1000	66
300003	124	0	6100	4300	1200	265	1073	4.87	yes	yes	75	10500	9900	600	293
300004	105	7	7100	6600	100	120	236	26.68	yes	no	86	6000	4800	300	142
300005	88	35	5100	4800	100	137	386	15.36	yes	no	102	4000	3000	500	140
100004	105	29	16100	15524	48	178	587	9.68	no	yes	78	6200	5237	187	138
300006	99	13	5100	5000	0	155	408	14.26	no	no	79	3100	2500	200	178
100005	86	19	5100	4804	36	110	703	7.23	yes	yes	74	2900	2351	169	89
300007	99	11	11100	10800	300	237	408	14.54	no	no	80	3500	2000	800	213
100007	90	4	10770	9995	86	212	577	10.38	no	no	96	8200	6995	500	308
100008	96	35	11570	11153	93	80	849	6.02	no	yes	81	3710	2755	622	90
100009	85	44	9010	8370	117	133	398	14.70	no	no	113	6030	5083	464	152
100010	76	38	17720	17224	106	96	271	21.63	yes	no	102	13050	12293	274	217
100011	83	25	8310	7911	83	91	152	42.92	yes	no	78	8530	7553	520	190
100012	102	8	16150	15391	48	401	491	11.26	no	no	89	10230	8501	409	386
100013	90	20	12640	12198	88	117	838	6.38	yes	no	93	6990	6123	245	169
100014	70	28	14340	13092	129	256	593	9.49	yes	no	87	8710	6672	444	389
300009	79	41	4600	4200	100	105	216	30.09	no	no	82	4000	3300	200	188
300010	65	78	7200	6300	400	141	554	9.69	yes	no	64	2300	1800	300	110
300012			4800	4600	100	148	552	9.98	yes	yes	66	2000	1600	100	97
300011	76	48	6500	6200	100	84	316	20.25	yes	no	80	4400		400	131
100015	74	30	10750	10050	20	218	917	5.79	yes	yes	77	9600	8170	710	256
100016	87	22	11200	10800	56	171	416	13.73	no	no	94	4070	3260	270	171
100017	97	33	9920	8800	210	139	415	13.63	no	no	96	6510	5130	630	167
100018	107	28	7100	6900	50	91	588	8.93	no	no	89	6600	5950	165	137
100019	81	9	11210	10430	90	308	353	16.58	yes	no	90	8200	7389	123	394
100020	73	36	7060	6700	113	107	451	11.99	yes	yes	82	3300	2360	600	151
100021	70	53	13940	13104	516	99	408	14.17	yes						
100022	81	19	5330	5069	53	79	685	7.40	no	no	91	5060	4250	258	101
100023	95	30	10800	10489	65	112	351	17.04	no	no	76	5020	4076	296	93
100024	82	40	4870	4388	161	151	586	9.26	yes	yes	89	7530	6001	745	200
100025	81	33	15020	14280	15	198	548	9.79	yes	no	84	8850	7133	903	256
300013	76	55	2300	1900	200	113	351	17.76	no	no	88	6900		230	213

Random Number	Re-Op	protocol biopsy day 7	rejection	clinical rejection banff classification	clinical rejection histology rating	protocol biopsy day 91	rejection	Banff classification	histology rating	protocl biopsy Day 181	rejection	Banff	histology rating
100001		yes	no	**Arterial intima fibrosis,C4d pos**	acute AbMR	no	n/a			yes	no		recurrence primary FSGS
300001		yes	no			yes	yes	**t1**,i0,g0,ptc0,v0/C4d neg	Borderline changes	yes	no		
100003		yes	no			yes	no			yes	no		
300002	yes	yes	no			yes	no			yes	no		
300003	yes	yes	no	t0,i0,**g1,PTC1**, v0/C4dneg	acute AbMR	no	n/a			yes	no		
300004	yes	yes	no			yes	no			yes	no		
300005		no	n/a			no	n/a			no	n/a		
100004		yes	no			yes	yes	t0,i0,**g1,ptc1,v1/C4d pos.**	acute AbMR	yes	yes	t0,i0,**g1**,**ptc1**,v0/C4d neg	acute AbMR
300006		no	n/a			yes	no			yes	no		
100005		yes	no			yes	no			yes	n		
300007		yes	no			no	n/a			yes	no		
100007		no	n/a			yes	yes	t1,i0,g0,ptc0,**v1**/C4d neg	IIA	yes	no		
100008		yes	no			yes	no			yes	yes	**t1,i1**,g0,ptc0,v0/C4d neg	Borderline changes
100009		no	n/a			yes	no			yes	no		
100010		no	n/a			yes	no			yes	no		
100011		no	n/a	**TMA**,t0,i0,g0,ptc0,v0/**C4d pos**	acute AbMR	yes	no			yes	no		
100012		no	n/a										
100013		no	n/a			no	n/a			yes	no		
100014		no	n/a			yes	no			yes	no		
300009		yes	no			yes	no			yes	no		
300010		no	n/a	t0,i0,g0,ptc0,**v1/C4d pos.**	acute AbMR	yes	no			yes	no		
300012		yes	no			yes	no			yes	no		IgA Nephritis
300011		yes	no			yes	no			yes	no		
100015		no	n/a	t0,i0,g0,ptc0,**v1**/C4d neg	IIA	yes	yes	t0,i0,g0,**ptc1**,v0/C4d neg	acute AbMR				
100016		yes	no			yes	yes	**g1**,ptc0,**v1**,i0,**t1**/C4dneg	acute AbMR	yes	no		
100017		yes	no			yes	yes	t0,i0,g0,**ptc2**,v0/**C4d pos**	acute AbMR	yes	yes	t0,i0,g0,**ptc2**,v0/**C4d pos**	acute AbMR
100018		no	n/a			no	n/a			yes	n		
100019		no	n/a			yes	no			yes	yes	**t1**,i0,g0,ptc0,v0/C4d neg	Borderline changes
100020		yes	no			no	n/a						
100021	yes												
100022		no	n/a			no	n/a			no	n/a		
100023		yes	no			yes	yes	**t1**,i0,g0,**ptc1**,v0/C4d neg	acute AbMR	yes	yes	**t1**,i0,g0,**ptc3**,v0/C4d neg	acute AbMR
100024		yes	no			no	n/a			no	n/a		
100025		yes	no			yes	no			yes	no		
300013		yes	no			yes	no			yes	no		

Random Number	Hb day 14	WBC day 14	Neutro day 14	Lympho day 14	Tc day 14	Hb day 21	WBC day 21	Neutro day 21	Lymph day 21	Tc day 21	Krea day 21	GFR day 21
100001	95	7350	6240	390	211	97	4700	3672	577	285	544	11.59
300001	73	5100	3500	900	278	94	4400	3500	900	200	205	34.10
100003	89	10600	9204	585	249	78	6600	5558	452	218	137	52.35
300002	88	11400	10000	700	155	77	8100	6700	900	218	133	49.37
300003	76	21300	19700	600	454	104	7700	6500	600	243	151	46.78
300004	76	11500	10000	800	363	76	11500	10000	800	363	123	56.59
300005	79	5200	ND	450	280	80	4300		420	153	230	27.91
100004	80	4900	4168	293	355	78	8000	6919	392	298	285	22.29
300006	79	4100	3500	300	263	87	3400			226	145	47.06
100005	75	6600	3050	148	184	88	4900	4585	108	228	353	16.00
300007	85	8500			491	97	11700			388	92	81.12
100007	104	14160	11625	1643	287	99	6630	4130	1797	282	167	43.42
100008	94	7600	6240	927	253	106	6490	5160	941	192	246	25.13
100009	116	8140	7204	586	234	115	6510	5599	566	209	150	45.33
100010	85	7460	6587	366	356	116	7840	6758	384	326	90	77.19
100011	88	10630	9163	829	142	88	6440	5641	399	281	107	64.36
100012	97	10320	9690	124	364	112	5710	4973	211	395	118	58.36
100013	93	20810	19749	291	442	94	8660	7768	251	194	125	57.37
100014	94	12110	10693	448	486	111	9040	8281	271	320	85	89.27
300009	100	4500	3800	300	260	100	4500	3800	300	260	102	71.54
300010	76	20000		1320	576	63	4800			243	176	36.39
300012	95	3700	2800	600	245	102	3000			240	95	76.06
300011	92	8400			265	99	5500			246	112	67.02
100015	83	11400	10200	760	391	86	9020	7387	1335	337	175	39.14
100016	80	8340	7164	492	437	91	6820	6124	246	379	160	41.37
100017	81	10830	9086	1105	434	100	5050	4010	555	193	169	38.42
100018	93	10960	10640	160	219	105	7570	6950	212	138	119	56.45
100019	80	11810	11326	189	416	85	9800	9251	206	266	94	76.33
100020	102	7400	6421	588	136	92	5280	4483	486	134	204	29.94
100021												
100022	93	6010	5361	240	81	94	3540	2945	262	80	124	53.19
100023	70	10030	8676	692	297	84	6690	5673	549	250	128	54.57
100024	81	6550	5273	832	411	80	5330	4541	624	393	263	23.34
100025	74	14430	11962	1804	422	96	9520	7949	1009	232	176	36.31
300013	75	8200			307	84	3900			265	199	34.19

Random Number	Hb day 28	WBC day 28	Neutro day 28	Lymph day 28	Tc day 28	Krea day 28	GFR day 28	Hb day 61	WBC day 61	Neutro day 61	Lymph day 61	Tc day 61	Krea day 61	GFR day 61
100001	110	5900	4606	864	259	274	25.58	124	4800	3514	895	201	378	17.65
300001	106	7000	5100	1300	179	193	36.56	127	7200	5300	1300	190	173	41.48
100003	109	9400	7290	1181	332	120	61.00	132	11500	9606	977	252	60	135.74
300002	91	5900	85500	11100	235	136	48.12	103	7900	6100	1300	216	150	42.98
300003	115	5800	5200	400	220	151	46.78	124	8600	8100	100	220	175	39.46
300004	114	8300	6700	1100	236	208	30.86	113	7200	5700	1100	231	117	59.95
300005	91	3700		420	156	207	31.52	118	4200			124	170	39.56
100004	84	4700	3612	447	340	225	29.28	109	8800	7647	580	255	162	42.77
300006	98	5500	4800	300	209	196	33.24	111	6500	5800	300	172	157	42.93
100005	107	4500	4005	162	170	187	33.31	124	4540	3963	173	122	107	63.44
300007	110	8700			377	77	99.62	143	7800	5300	1800	323	64	123.32
100007	111	8630	5618	2252	357	193	36.75	114	8980	5707	1994	226	207	33.89
100008	107	5180	3833	1036	152	193	33.24	127	6780	5010	1207	117	175	37.22
100009	123	5590	4299	922	164	126	55.43	129	9690	8673	562	204	145	47.14
100010	112	7450	6429	402	268	93	74.33	127	10790	9269	669	333	82	85.95
100011	98	7570	7078	197	221	98	71.23	131	3360	2980	165	295	85	83.94
100012	112	7310	6637	234	323	113	61.35	135	12740	11772	344	248	119	57.80
100013	116	8940	8207	250	179	117	61.92	135	13920	13085	237	179	126	56.85
100014	126	10420	9513	344	287	88	85.77	133	14500	13007	537	312	99	74.87
300009	96	3900			206	87	85.95	118	4600			181	99	74.04
300010	78	7100		260	349	116	58.88	91	4500	3700	400	311	116	58.88
300012	111	3300	2500	700	173	88	83.08	129	6400			204	117	59.81
300011	108	5600		910	210	113	66.33	128	6700			197	116	64.36
100015	89	8000	6624	944	278	169	40.75	112	9260	7325	1380	275	116	62.91
100016	101	6680	5725	401	279	143	47.10	115	6650	5433	612	264	158	41.97
100017	93	6240	4992	724	202	133	50.66	105	4840	3741	610	228	155	42.46
100018	101	6040	5170	420	324	114	59.32	103	5690	5250	148	128	118	57.00
100019	95	9440	8760	245	241	91	79.25	107	9260	8269	407	247	113	61.72
100020	83	2410	1470	665	154	127	51.74	96	4740	3670	740	159	116	57.44
100021														
100022	106	2620	2075	288	69	115	58.02	116	3200	2672	301	58	106	63.74
100023	97	5370	4258	650	194	127	55.07	120	5980	4545	897	246	133	52.21
100024	90	4960	3973	729	335	151	44.27	96	4050	3118	668	196	199	32.19
100025	89	6630	5198	981	220	172	37.29	114	9350	7246	1234	269	224	27.49
300013	104	5400		330	214	166	42.14	124	5400			210	153	46.30

Random Number	Hb day 91	WBC day 91	Neutro day 91	Lymph day 91	Tc day 91	Krea day 91	GFR day 91	Prot/crea day 91
100001	93	4500	3125	827	162	216	33.66	254
300001	133	7800	5800	1200	211	166	43.50	45.71
100003	134	11000	9789	547	254	54	158.98	
300002	109	6900	5300	1300	199	164	42.54	35.09
300003	107	6800	6300	200	238	200	30.83	56.12
300004	128	5500	4200	900	208	115	64.05	30.08
300005	123	5200		780	131	187	34.89	12.52
100004	113	7010	5973	442	217	177	37.76	8
300006	101	5600	4900	400	312	204	32.78	45.87
100005	128	5410	4642	276	138	109	65.42	39
300007	154	7700		1188	314	62	119.08	17.05
100007	118	9310	6889	1694	233	186	36.00	14
100008	134	6550	4919	1153	102	151	48.78	9
100009	141	8060	6811	758	156	121	56.98	5
100010	144	11800	9816	648	282	79	95.00	5
100011	136	3950	3527	205	239	93	74.33	22
100012								
100013	144	5270	4580	179	161	132	51.28	27
100014	141	7970	6759	582	202	98	75.97	7
300009	129	6500	5100	700	190	99	74.87	16.84
300010	112	3200	2000	600	193	94	78.61	33.22
300012	138	5600		410	217	124	54.51	28.85
300011	140	5100		1111	202	124	55.93	11.18
100015	115	5920	4280	1231	305	111	67.71	11
100016	116	8710	7386	653	293	164	42.19	18
100017	121	4520	3625	434	193	122	56.57	29
100018	105	7320			125	198	32.01	
100019	114	7630	6646	435	249	127	52.37	9
100020	127	12060	9760	1140	214	113	61.72	
100021								
100022	113	2970	2462	291	56	97	74.36	
100023	129	4740	3351	820	257	116	57.44	107
100024	101	3280	2424	600	157	169	39.60	96
100025	114	8230	6107	1226	294	198	32.38	22
300013	126	4700	3900	500	197	130	51.51	15.08

Random Number	Hb day 181	WBC day 181	Neutro day 181	Lymph day 181	Tc day 181	Krea day 181	GFR day 181
100001	77	4200	2341	1025	213	300	23.04
300001	124	9500			190	182	39.12
100003	130	12900	11167	904	285	68	117.48
300002	92	6600			209	145	44.69
300003	114	7700	7400	100	215	204	33.06
300004	123	2800	1700	800	234	116	60.55
300005	125	2600		590	152	179	37.28
100004	100	4630	3593	500	297	204	32.78
300006	109	3300	2300	400	193	157	42.93
100005	141	6180	5166	457	117	104	65.55
300007	125	5100		1080	321	66	119.02
100007	115	6640	4402	1660	222	224	30.94
100008	131	6550	4821	1218	121	152	43.79
100009	150	9400	7896	780	162	111	64.16
100010	135	8120	6772	593	241	83	84.75
100011	122	4030	3462	318	250	101	68.79
100012							
100013	150	10560	9536	348	164	138	51.18
100014	132	7280	5962	648	310	116	62.36
300009	125	3900			167	88	84.82
300010	109	4600			162	103	67.53
300012	142	5400		900	177	103	69.28
300011	133	3800		720	208	121	61.30
100015							
100016	121	8640	7370	631	219	186	34.77
100017	106	3620	2853	427	181	127	53.43
100018	129	3160			131	211	29.15
100019	108	7430	6754	312	272	137	49.42
100020							
100021							
100022	115	3700	2956	422	73	108	62.38
100023	121	4500	3312	657	233	117	60.53
100024	116	2120	956	903	162	154	43.28
100025	110	9580	7060	1255	325	184	34.50
300013	127	3900			194	144	49.66

Prot/crea day 181	Hb day 365	WBC day 365	Neutro day 365	Lymph day 365	Tc day 365	Krea day 365	GFR day 365	Prot/crea day 365	Krea day 730	GFR day 730	Prot/crea day 730	CD3 day 1	CD19 day 1	CD3 day 180	CD19 day 180	CD3 day 365	CD19 day 365	date of recipients death
346	162	8360	6128	1070	190	187	39.47	146	211	34.11	667	16	58	543	262	440	169	
34.43	-	-	-	-	-	-												Exitus letalis 04.01.2010
7	151	9300		1650	231	64	125.41	5	61	131.94	11							
50	116	5900	-	1920	174	132	49.66	161	172	36.48	45.45	0.87	0.17	0.86		1.54	0.11	
40	145	6100	-	220	202	172	40.06	103	287	22.09	359.74	1.14	0.13	0.05	0.02	0.13		
11.03	130	3700	-	770	199	110	64.13	0.06	110	63.90	12.68	0.87	0.04	0.66	0.04	0.61	0.03	
18.35	113	3700	-	-	156	158	42.88	12	175	37.96	11.83	1.00	0.10	0.39	0.07	0.54	0.07	
6	116	5530	4385	476	230	186	36.30	5	178	38.02	3			265	8	377	1	
43.68	124	5400	-	1090	265	139	49.22	30	160	41.69	35.47	0.47	0.03	0.41	0.02	0.92	0.04	
129	148	7430	6390	453	128	96	71.68	35	84	83.38	16	7	4					
14.08	124	4900	-	1118	202	79	96.31	13			12.64	0.13	0.29	0.67	0.31	0.88	0.21	
6	129	7250	4945	1689	210	178	40.11	11	182	38.88	6	4	67	1002	140	1377	142	
8	137	7570	5503	1363	126	141	47.59	7	143	46.66	7							
7	146	10100	8100	1101	189	110	64.59	9	112	63.02	16	16	71					
19	134	7910	6644	570	259	77	92.15	8	87	79.81	13	19	65					
18	125	2350	1610	489	289	91	77.34	25	106	64.65	10							
												11	32					Exitus letalis 08.11.2010
17	168	11970	10378	551	186	154	44.90	16	131	53.88	12	9	61					
7	141	9160	7200	788	324	117	61.48	4	113	63.73	7	8	48	486	26	584	37	
21.64	131	4000	-	800	178	91	81.27	15	105	68.62	16.15	0.01	0.04	0.63	0.07	0.50	0.06	
26.35	112	4500	-	1350	187	81	88.82	10	59	127.63	ND	1.21	0.20	0.54	0.00	1.24	0.03	
22.37	135	3400	-	700	152	119	58.43	21	119	58.22	14.63	0.82	0.04	0.77	0.04	0.60	0.02	
23.97	139	8100	-	-	369	113	66.00	96	104	72.28	15.22	1.41	0.13	0.52	0.09	0.60	0.09	
												25	108					Exitus letalis 22.10.2011
11	116	6450	4670	1019	312	172	37.92	12	204	31.03	29	5	12	514	73	827	56	
30	109	4330	3105	628	200	149	44.29	22	242	25.22	7	56	182	258	233	271	196	
	130	6820	5838	430	118	211	29.06	22	252	23.60	18	9	54			260	102	
5	115	7810	7029	234	226	123	55.77	16	117	58.88	7	3	19			169	7	
												60	71					Exitus letalis 26.05.2012
																		Exitus letalis 15.06.2012
9	110	2720	2241	234	50	108	62.20	9	120	54.92	10							
60	143	5710	3757	1022	224	164	40.84	27	125	55.65	24	30	80	592	131	768	118	
59	142	5880	3499	1764	155	162	40.67	39	169	38.60	30			806	60	1512	112	
18	110	6710	3872	1543	329	118	57.42	14	118	57.23	57			553	467	396	258	
13.86	143	5200	-	540	176	124	58.73	0.06	149	47.29	1.40	0.81	0.08	0.37	0.07	0.34	0.06	
